# Prediction of Activity and Selectivity Profiles of Sigma Receptor Ligands Using Machine Learning Approaches

**DOI:** 10.1021/acs.jcim.5c01091

**Published:** 2025-09-01

**Authors:** Lisa Lombardo, Verena Battisti, Thierry Langer, Rosaria Gitto, Laura De Luca

**Affiliations:** † Department of Chemical, Biological, Pharmaceutical and Environmental Sciences (CHIBIOFARAM), University of Messina, Viale Ferdinando D’Alcontres 13, I-98166 Messina, Italy; ‡ Department of Pharmaceutical Chemistry, 27258 University of Vienna, Althanstraße 14, A-1090 Vienna, Austria

## Abstract

Sigma (σ) receptors (SRs) have emerged as important therapeutic targets due to their roles in various biological pathways. They are classified into two subtypes: S1R, primarily distributed in the central nervous system and related to neuroprotection and neurodegenerative diseases, and S2R mainly expressed in cancer cells and associated with cell proliferation and apoptosis, as well as in neurons. Although S1R and S2R exhibit structural differences in receptor architecture and assembly, they share similar binding site features and ligand recognition mechanisms. This similarity underscores the importance of identifying selective ligands for therapeutic design, especially given the distinct physiological functions of these receptors. In this project, we developed three distinct machine learning (ML) approaches based on classification, regression, and multiclassification models to predict the activity and selectivity profiles of SR ligands. High-quality data sets were curated from public and in-house source; in turn, the data sets were systematically organized and processed for each workflow. Models were built using molecular descriptors and fingerprints, including Mordred, RDKit, ECFP4, ECFP6, and MACCS keys, and trained with various ML algorithms such as extra trees, random forest, support vector machine, *k*-nearest neighbors, and XGBoost. Rigorous nested and classical 5-fold cross-validation protocols were applied for model selection and validation. At the end, identification of the best workflow was performed by an external validation procedure. Among the workflows, the one-step multiclassification approach, based on extra trees combined with Mordred descriptors, showed the best predictive performance in external validation, offering a robust tool for the identification of selective S1R and S2R ligands.

## Graphical abstract

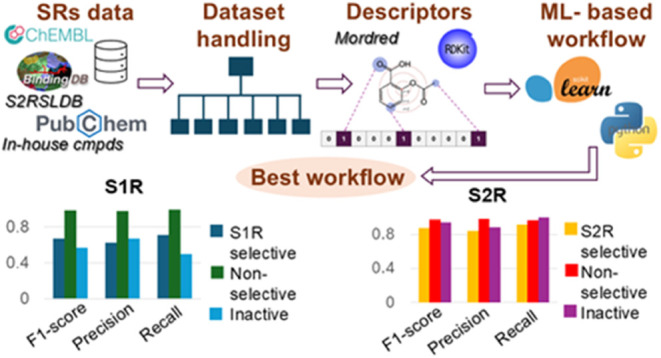

## Introduction

Sigma (σ) receptors (SRs) were initially classified as subtypes of opioid receptors; they were subsequently defined as nonopioid receptors having peculiar mechanisms of signal transduction acting as membrane proteins that regulate cellular transmission affecting the expression level of distinct receptors and transporters in both nervous system and peripheral tissues. S1R receptor consists of a small transmembrane protein mainly localized in the mitochondria-associated endoplasmic reticulum (ER). It was well established that S1R interacts with ion channels and G-protein coupled receptors, thus exerting a chaperone role in several neurological diseases as well as cancer progression.^1–4^ The S2R is overexpressed in tumor cells and regulates their proliferative status;^5^ furthermore, S2R shows increased expression in neurons adjacent to Aβ oligomers in patients with Alzheimer’s disease (AD).^6^ Recently, S2R has been structurally identified as the transmembrane protein of the endoplasmic reticulum (ER), that is, the cholesterol-responsive NPC1-binding protein TMEM97.^7^

Despite S1R and S2R not being homologues, their binding pockets possess a certain similarity and overlap in ligand recognition mapping. Notably, they have been shown as clinically relevant biomarkers in neurological disorders and in a wide variety of tumors including gastric, pancreatic, colorectal, breast, prostate, and lung cancer.^5,8–13^ Therefore, SRs ligands offer multiple therapeutic applications in human diseases so that significant efforts have been made to identify selective ligands acting as agonist/antagonists, thus exerting a role in regulating the chaperone activity of both S1R and S2R subtypes.^14,15^ Despite significant advances in the study of SRs, distinguishing between agonists and antagonists for both S1R and S2R subtypes remains unfeasible due to insufficient experimental data collected by chemical databases. Notably, functional assays for S2R remain relatively underdeveloped and are often indirect, which continues to hamper comprehensive functional characterization.^16^ Given these limitations, our research has prioritized predicting the activity and selectivity of compounds targeting SRs as a relevant potential for the development of novel therapeutic strategies.

Computational methods, including quantitative structure–activity relationship (QSAR) models and machine learning (ML) techniques, have often been employed to predict compound selectivity toward the distinct protein targets.^17–22^ For example, Tinivella et al. developed binary classification models to predict selective inhibitors for carbonic anhydrase (CA) isoforms II, IX, and XII, determining selectivity profiles based on predicted active and inactive labels of ligands.^17^ Recently, Djokovic and co-workers have introduced a machine learning-based tool named SIRT2i_Predictor, investigating binary, multiclass, and regression models to predict selective sirtuin 2 (SIRT2) inhibitors.^18^ Burggraaff et al. constructed quantitative selectivity models for adenosine receptor subtypes A_1_ and A_2A_, directly training on the affinity differences of ligands for these subtypes.^19^ The ML approaches mentioned above have been efficaciously applied to model selectivity of ligands targeting other protein families, yielding promising results; similarly, in this study, we developed and assessed three different ML-based workflows to predict the activity and selectivity profiles for SRs ligands.

Particularly, we aimed to develop ML models capable of predicting the activity and selectivity profiles of SRs compounds using a variety of molecular descriptors. Five distinct classification and regression ML algorithms were employed to construct models, each leveraging five different types of molecular descriptors. ML models were trained and evaluated using a nested 5-fold cross-validation approach to ensure robust and unbiased performance. The best-performing models were identified and fine-tuned through a traditional 5-fold cross-validation process.

Finally, to determine the most effective workflow, the selected ML models were tested and validated against previously unseen data, i.e., the external validation data set. To compare the three workflows, metrics such as area under the receiver operating characteristic curve (ROC-AUC) and Matthews correlation coefficient (MCC) scores were employed alongside detailed classification reports that included precision, recall, and *F*1-score for each labeled class.

## Results and Discussion

The focus of this work was to develop QSAR models and ML techniques to design reliable workflows to predict the activity and selectivity of S1R and S2R ligands. In detail, the workflows were tailored to the specific tasks: (i) a 2-step workflow utilizing binary classification models (Figure 1A), (ii) a 2-step workflow based on regression models (Figure 1B), and (iii) a 1-step workflow employing multiclassification models (Figure 1C), referred to as 2-step classification workflow, 2-step regression workflow and 1-step multiclassification workflow, respectively. This approach allowed us to assess various modeling techniques and identify the most effective strategy for our objectives. The 2-step workflows, involving separate models for activity prediction and selectivity assessment, could provide flexibility in model selection and optimization for each task. This separation enables us to fit models into the unique activity and selectivity data features, potentially enhancing the predictive performance. Conversely, the 1-step multiclassification workflow aims to predict activity and selectivity within a single model simultaneously. This integrated approach offered the advantage of capturing the interdependence between activity and selectivity, which could lead to more accurate predictions.

**Figure 1:**
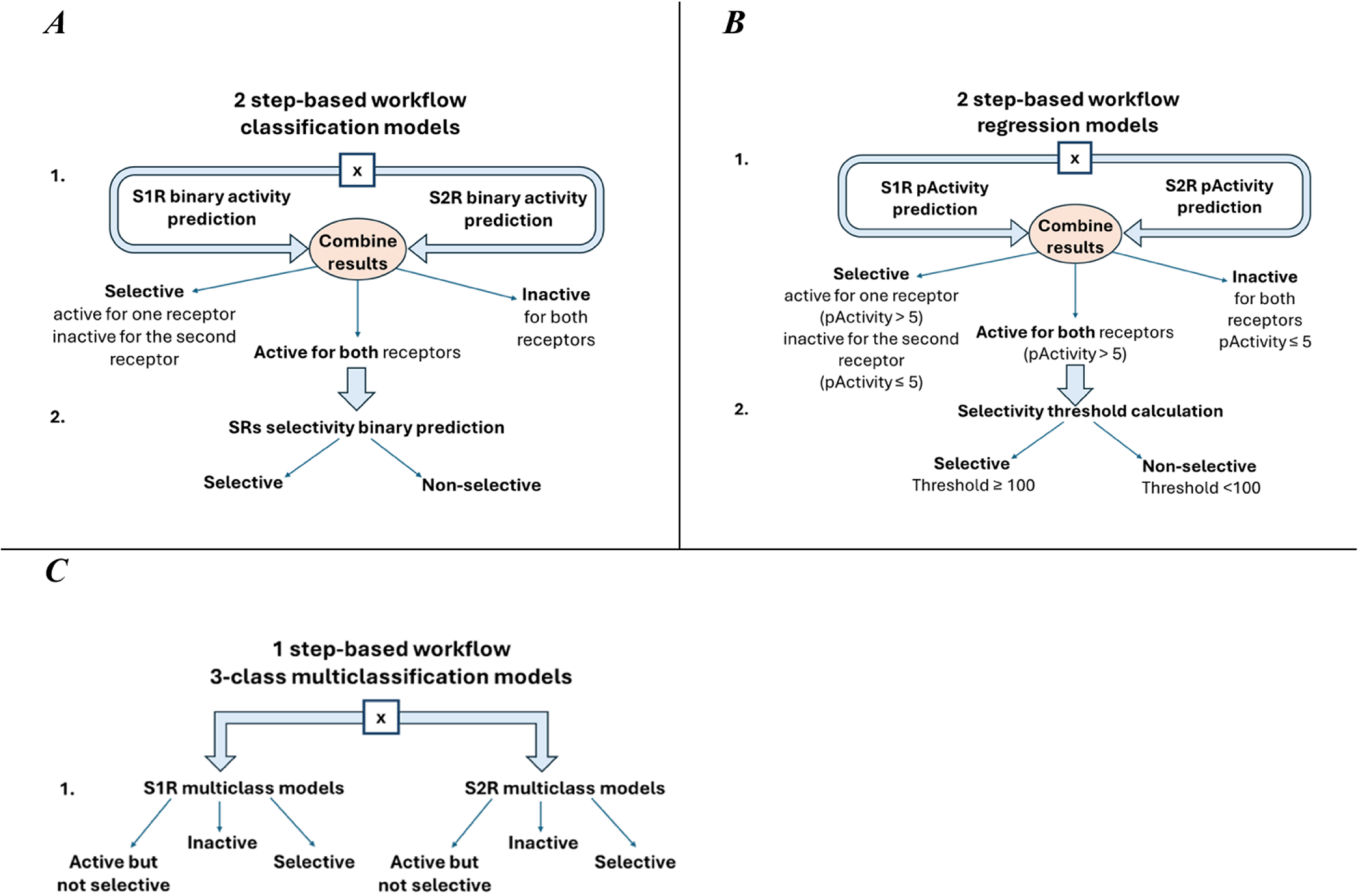
Schematic of proposed workflows to predict activity and selectivity for ligands targeting S1R and S2R.

By implementing these distinct workflows, we aimed to comprehensively evaluate the strengths and limitations of each modeling strategy, ultimately selecting the most effective approach for predicting SR ligand profiles. Hence, ML models were developed and trained using 2D molecular descriptors, including Mordred^23^ and RDKit^24^ (RDKit: Open-source cheminformatics; https://www.rdkit.org/), as well as molecular fingerprints such as Morgan fingerprints with radius 2 and 3 (corresponding to ECFP4 and ECFP6) and MACCS Keys.^25,26^

To facilitate these workflows, a curated data set of bioactivity records for SRs was assembled. This data set included compounds demonstrating affinity for S1R and S2R, the biological data were collected from public databases such as ChEMBL,^27^ BindingDB,^28^ PubChem BioAssays,^29^ and S2RSLDB,^30^ supplemented by an in-house data set of previously reported compounds.^31,32^ The search was carried out using two selected UniProt ID keywords: Q99720 for S1R and Q5BJF2 for S2R. The raw data set was merged and filtered to retain only activity records expressed as *K*_i_, IC_50_, *K*_d_, and EC_50_ values, resulting in a preliminary and uncleaned collection of bioactivity rows.

Subsequently, a comprehensive data curation process was applied, as described in the Materials and Methods section. This process included standardizing molecular representations as canonical SMILES, removing salts, eliminating duplicates, and converting bioactivity measures into standardized pActivity values. After this rigorous curation, the data set consisted of 4994 compounds and 6110 high-quality activity records, that were based on *K*_i_ value for 84.3% of collected compounds, IC_50_ value for 15.53% of collected compounds and *K*_d_ value only for 0.19% of collected compounds. The gathered compounds were classified according to their activity and selectivity profiles (Figure 2A). In more detail, we defined compounds as “active” if they possessed a *K*_i_, IC_50_, and/or *K*_d_ values <10,000 nM (pActivity >5). Conversely, we classified compounds with a *K*_i_, IC_50_, and/or *K*_d_ ≥ 10,000 nM as “inactive” (pActivity ≤ 5) in accordance with data reported in the literature.^31,33–35^

**Figure 2:**
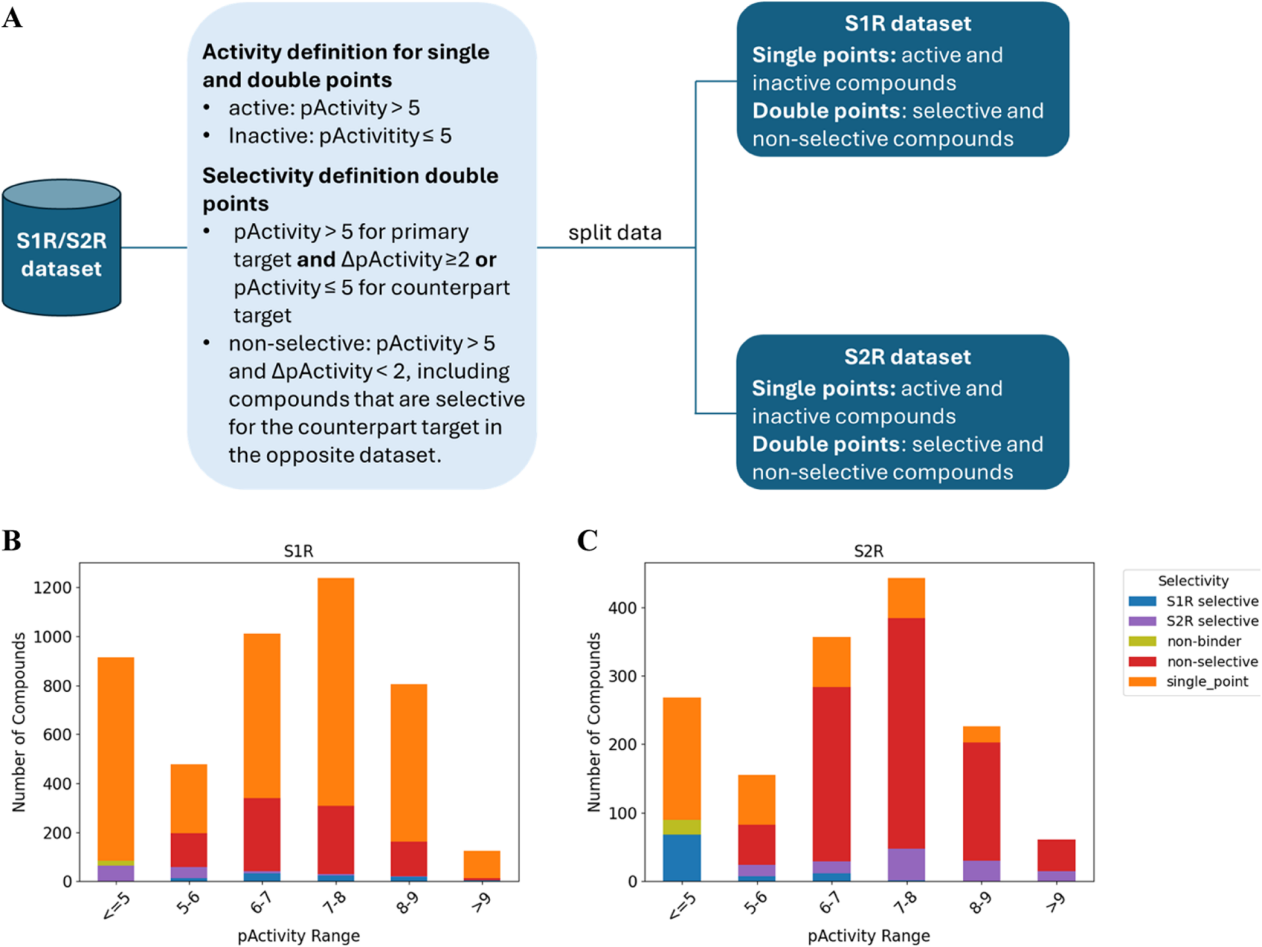
Schematic representation of data point definitions, including activity and selectivity criteria used to classify compounds within each data set. Distribution of activity data for S1R (B) and S2R (C). The *x*-axis indicates the pActivity range, while the *y*-axis corresponds to the number of compounds. Compounds possessing activity records for only one target are referred to as single points. In this case, only activity definition is possible, i.e., active when pActivity is >5 and inactive when pActivity is ≤5. Double-point compounds are classified as selective when pActivity is >5 and ΔpActivity ≥2 or pActivity ≤5 for the counterpart target, nonselective if pActivity is >5 for both protein and ΔpActivity <2. Additionally, in the S1R data set, compounds selective for S2R were labeled as nonselective, and vice versa for the S2R data set. Compounds with pActivity ≤5 for both targets were classified as nonbinders.

Additionally, for compounds that demonstrated to be active toward both receptors, so-called “double points”, the selectivity profiles were assigned based on the following criteria: “S1R-selective” if pActivity >5 (i) and the activity was ≥100-fold higher than S2R (i.e, ΔpActivity ≥2) (ii) or when was inactive for S2R; conversely, “S2R-selective” when pActivity >5 (i) and the activity was ≥100-fold higher than S1R (i.e, ΔpActivity ≥2) (ii) or inactive for S1R. Compounds active for both targets (pActivity >5) and with less than a 100-fold difference in activity were labeled as “nonselective” (i.e ΔpActivity <2). Additionally, in the S1R data set, compounds selectively targeting S2R were assigned to the nonselective class, and the same criterion was applied in reverse for the S2R data set. Compounds that were inactive for both targets were categorized as “nonbinders”. For molecules for which we found biological data measured for only one receptor (i.e, “single-point” compounds), we were not able to assign a specific selectivity category. The distribution of activity data for S1R and S2R is reported in Figure 2B,C, respectively.

Compounds in the data set were strategically handled to train different models based on the main task. First, the external validation set, corresponding to 10% of S1R-selective, S2R-selective, nonselective, and nonbinder classes, was extracted to ensure a proper comparison of binary classification, multiclass classification, and regression models within the three workflows. Indeed, the external data set remained untouched by model training or testing, providing an unbiased measure of model performance. Using compounds with data for both receptors (i.e., double points) was mandatory to guarantee that all of the key classes were represented in the external validation set, especially for selectivity prediction. The final external validation set comprised 220 data points corresponding to 110 “double-point” compounds (i.e., molecules with activity data for both S1R and S2R): 88 nonselective, 8 S1R-selective, 12 S2R-selective, and 2 nonbinder compounds. To carefully assess the independence from the training data, we performed similarity and chemical space analysis. Therefore, we calculated the maximum ECFP4-based Tanimoto similarity between each external compound and all double-point training compounds. The mean and median of these maximum similarities were 0.70 and 0.72, respectively, while only 22% of external compounds exceeded a similarity threshold of 0.85, therefore, indicating substantial chemical diversity. Further, a t-SNE projection of ECFP4 fingerprints (Supporting Information, Figure S1) shows that, despite some overlaps due to random sampling, many test compounds, especially selective ligands, occupy sparsely populated or unique regions of chemical space. This evidence supports the external set as a valid and nonredundant benchmark for model evaluation. Subsequently, a series of different data sets were built to develop ML models within each workflow (Table 1 and Figure 3). For the two-step-based workflows, Data set 1 and Data set 2 were built, including all collected data for S1R and S2R, respectively. Notably, the first phase involves the prediction of activity toward the two receptors as a binary classification model to predict activity labels or as pActivity prediction in the case of a regression task. Data set 1 was the largest among all data sets, comprising 4476 compounds (3569 active and 907 inactive). In contrast, Data set 2 contained fewer compounds due to the limited data available for S2R, encompassing only 1414 derivatives (1154 active and 260 inactive).

**Figure 3:**
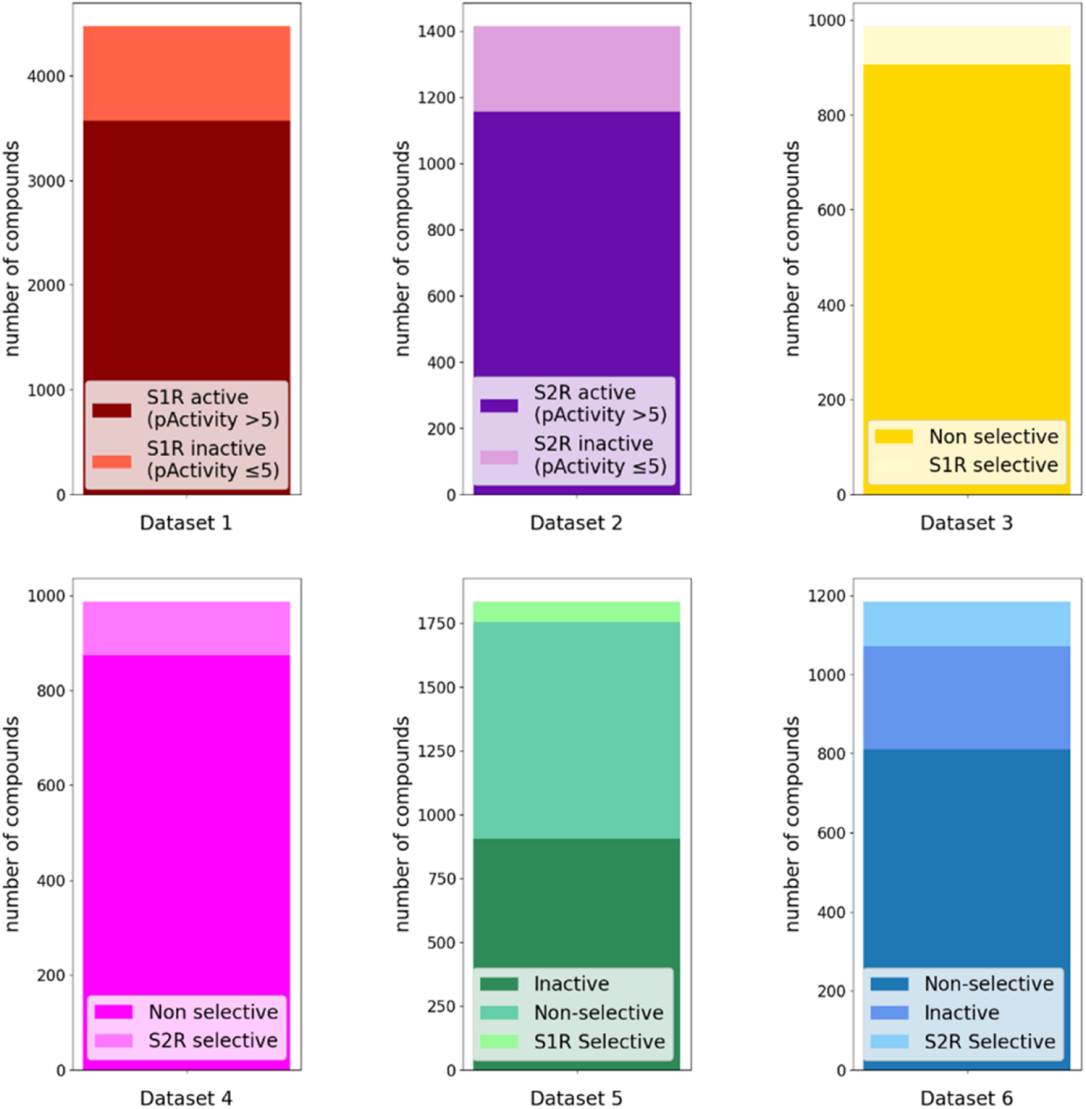
Distribution of data within each data set. Data sets 1 (red) and 2 (purple) were used to predict activity labels and pActivity values, while Data sets 3 (yellow) and 4 (magenta) were used to predict selectivity labels. Data sets 5 (green) and 6 (blue) were designed to predict multiclass labels. Specifically, Data sets 1 and 2 included compounds with activity data for S1R and S2R, respectively. Data sets 3 (yellow) and 4 (magenta) encompassed only double-point compounds with selectivity definitions for S1R and S2R, respectively. Finally, Data sets 5 and 6 contained double-point compounds for selectivity definition and single-point inactive compounds for S1R and S2R, respectively.

**Table 1:** Description of Data Sets Reporting Composition, Number of Compounds, and Average Pairwise Tanimoto Similarity within Each Data Set, Calculated Using ECFP4 Fingerprints

data set	composition	number of cmpds^a^	avg similarity
1	compounds with activity data for S1R	4476	0.130
2	compounds with activity data for S2R	1414	0.146
3	double points, including S1R-selective and nonselective	987	0.158
4	double points, including S2R-selective and nonselective	987	0.158
5	double points (encompassing S1R-selective, nonselective, and nonbinders) and inactive single points	1835	0.116
6	double points (encompassing S2R-selective, nonselective, and nonbinders) and inactive single points	1185	0.148

a Compounds possessing biological data.

As for selectivity prediction by binary classification models, Data Sets 3 and 4 were generated. Notably, the two data sets include the same data points: S1R-selective, S2R-selective, and nonselective, resulting in 987 compounds. The two data sets differ in the labels assigned to the compounds. To predict S1R selectivity, Data set 3 encompassed 81 S1R-selective and 906 S1R nonselective molecules. Conversely, for the prediction of S2R-selective ligands, Data set 4 included 114 S2R-selective and 873 S2R nonselective compounds.

Concerning three-multiclass models, Data sets 5 and 6 were designed to predict selective, nonselective, and inactive compounds. In this case, except for the inactive class, we needed to use compounds with data for both receptors to train and test the multiclass models. Data set 5 was composed of S1R-selective (81 compounds), S1R nonselective (906 compounds), and S1R inactive (907 compounds), while Data set 6 included S2R-selective (114 compounds), S2R nonselective (873 compounds), and S2R inactive (260 compounds). To assess the internal chemical diversity of each data set, we calculated the mean pairwise Tanimoto similarity (Tc) between compounds within each set using ECFP4 fingerprints (Table 1). The data sets showed low average Tc values (0.116–0.158), indicating high structural diversity and low redundancy. This supports the suitability of the data sets for training generalizable ML models.

Molecules in each data set were described by calculating RDKit and Mordred 2D molecular descriptors. Furthermore, Morgan (or ECFP4 and ECFP6) and MACCS key fingerprints were calculated to investigate whether choosing different fingerprints influenced the outcome. For nested and classical 5-fold cross-validation protocols, data sets were divided into 80% for the training and 20% for the test set.

Considering the severe imbalance issue for classification tasks, Data sets 3–6 were submitted to an undersampling procedure to avoid any bias in model prediction and to prioritize the minority class for training the classification models.

### Nested Cross-Validation and Model Selection

In this study, various regression, binary, and multiclass classification models were developed by combining 5 ML algorithms, i.e., random forest (RF), extra trees (ET), extreme gradient boosting (XGBoost), support vector machines (SVM), and *k*-nearest neighbors (*k*NN), with five types of descriptors and fingerprints: RDKit descriptors, Mordred descriptors, ECFP4, ECFP6, and MACCS key fingerprints. All these molecular representations denote a well-established and straightforward approach in chemoinformatics to describe molecule patterns.^36^

The primary objective was to identify the best models and molecular descriptors for addressing regression, binary, and multiclass tasks. To this end, nested cross-validation was performed for each ML algorithm–descriptors combination. In the outer loop of the nested cross-validation (referred to as the “outer test fold”), we assessed model performance, while in the inner loop, we performed hyperparameter tuning. This procedure was repeated five times, rotating the outer folds.

We first employed nested cross-validation to obtain an unbiased selection of hyperparameters across multiple outer folds. Those parameters that consistently showed strong performance were then held fixed during the subsequent classical 5-fold cross-validation, while any remaining hyperparameters were tuned over a narrower range. This strategy refines only parameters inconsistent across folds, while preserving the primary tuning results established by nested cross-validation. It thus avoids redundant tuning, preserves key insights from nested cross-validation, and maintains a clear separation between model selection (nested cross-validation) and final performance estimation (classical cross-validation).

In nested cross-validation, the goal is not necessarily to select a single best model across all outer folds but to evaluate the model’s generalization performance on unseen data. Each outer test fold yielded a performance estimate for the model trained on its corresponding training portion (with hyperparameter tuning in the inner loop). These models were not merged into a single “ultimate” model; instead, we reported the average performance across all outer folds to characterize each algorithm-descriptor combination. After nested cross-validation, two criteria identified the most accurate ML models: average performance (unbiased effectiveness estimate) and standard deviation (result consistency across folds).

Simultaneously, we compared the performance of each model to determine the most suitable molecular descriptors, identifying which descriptor types best characterized the data and enhanced the predictive power.

### Binary Classification Models

In the 2-step classification workflow (Figure 1A), the prediction of activity and selectivity was approached as two sequential binary classification tasks. First, models were developed to differentiate active compounds from inactive ones for both S1R and S2R. Subsequently, binary classification models were constructed to distinguish selective compounds from nonselective ones. Multiple models were implemented to predict both activity and selectivity for the S1R and S2R subtypes, enabling a comprehensive evaluation of the predictive performance across these tasks. Binary classification models were developed using Data set 1 and Data set 2 to predict the activity status (active or inactive) of compounds for S1R and S2R. Data sets 3 and 4 were utilized to differentiate between selective and nonselective compounds. The nested cross-validation results for the S1R activity prediction indicate that both XGBoost and ET models utilizing Mordred descriptors emerged as the most robust and reliable approaches. These models demonstrated consistently high performance across multiple metrics, showcasing their effectiveness and stability. Specifically, in the outer test folds, the XGBoost model achieved an ROC-AUC of 0.973 ± 0.006, an *F*1-score of 0.954 ± 0.006, a precision of 0.975 ± 0.004, a recall of 0.933 ± 0.009, and an MCC of 0.794 ± 0.024 (Supporting Information, Table S1A). Similarly, the ET model exhibited comparable results with an ROC-AUC of 0.971 ± 0.007, an *F*1-score of 0.954 ± 0.005, a precision of 0.971 ± 0.005, a recall of 0.937 ± 0.009, and an MCC of 0.790 ± 0.023 (Table S1A). These findings underscore the robustness and predictive power of both models, with only slight variations across metrics, suggesting a high level of stability and reliability in predicting S1R activity. A direct comparison of foldwise ROC-AUC scores between the two models yielded a *p*-value of 0.0625, indicating that the difference was not statistically significant, although close to the conventional threshold of 0.05 (Table S1B). Concerning the RF Mordred model, a comparable ROC-AUC score (0.972 ± 0.005) was detected. Nevertheless, performance decreased when other metrics, such as the *F*1-score, recall, and MCC, were observed (Table S1A). In contrast, the outer test folds models like *k*NN and SVM showed higher variability and lower performance, making them less reliable for activity prediction. In all cases, quality metrics highlighted Mordred 2D descriptors as the best-performing molecular descriptors for model training within this project (Table S1A).

Regarding the S2R activity prediction, a general decrease in metrics performance is observed, probably due to the lower amount of data compared to Data set 1 (Table S2). Again, among the molecular representations, Mordred descriptors provided the best results in activity prediction (Table S2). Unlike results collected for S1R, ET (ROC-AUC = 0.892 ± 0.030, *F*1-score = 0.878 ± 0.023, precision = 0.953 ± 0.010, recall = 0.815 ± 0.039, and MCC = 0.542 ± 0.050) and SVM models (ROC-AUC = 0.882 ± 0.043, *F*1-score = 0.880 ± 0.033, precision = 0.953 ± 0.017, recall = 0.817 ± 0.050, and MCC = 0.544 ± 0.087) achieved the best performance across outer test folds (Table S2). Nevertheless, for the SVM model, higher standard deviation values highlighted variability across folds, reducing its consistency. For this reason, ET using Mordred descriptors was selected for classical 5-fold cross-validation (Table S2).

The second step of the two-step classification workflow focused on predicting selectivity for S1R and S2R using Data sets 3 and 4 for model training and testing. Notably, a general trend emerged in selectivity prediction: models utilizing ECFP4 fingerprints for S1R and ECFP6 fingerprints for S2R achieved the best performance (Tables S3 and S4). This finding suggests that these specific fingerprints effectively capture the variability within the data sets, enabling more accurate training of models for selectivity classification. Among the five models evaluated, the SVM model demonstrated superior performance in predicting selectivity for S1R, achieving an ROC-AUC of 0.881 ± 0.027, an *F*1-score of 0.471 ± 0.114, a precision of 0.387 ± 0.127, a recall of 0.629 ± 0.127, and an MCC of 0.429 ± 0.122 across outer test folds (Table S3). Similarly, for S2R, the SVM algorithm utilizing ECFP6 fingerprints outperformed the other models, with an ROC-AUC of 0.884 ± 0.058, an *F*1-score of 0.545 ± 0.078, a precision of 0.425 ± 0.087, a recall of 0.772 ± 0.078, and an MCC of 0.496 ± 0.091 (Table S4).

### 3-Class Multiclassification Models

Several models were built to predict both activity and selectivity for the SR subtypes in one step. The five multiclass models were developed using Data sets 5 and 6 to predict selective, nonselective, and inactive compounds for S1R and S2R, respectively. Based on the nested cross-validation results for S1R and S2R prediction, ET using Mordred descriptors emerged as the most robust model, demonstrating high performance and stability across quality parameter metrics, reporting the following quality metrics for S1R multiclassification: ROC-AUC = 0.941 ± 0.012, an *F*1-score 0.852 ± 0.026, precision = 0.881 ± 0.023, recall = 0.836 ± 0.025, and MCC = 0.718 ± 0.042 (Table S5), and for S2R multiclassification: ROC-AUC = 0.870 ± 0.031, an *F*1-score 0.766 ± 0.020, precision = 0.811 ± 0.028, recall = 0.751 ± 0.018, and MCC = 0.719 ± 0.045 across outer test folds (Table S6). The XGBoost and RF models demonstrated strong performance with a high degree of overlap in their results but were slightly outperformed by the ET models (Tables S5 and S6). In contrast, models such as *k*NN and SVM exhibited greater variability and lower performance, rendering them less reliable for prediction tasks. Among the molecular representations, Mordred descriptors provided the most detailed structural information, yielding the best results. Ultimately, ET models using Mordred descriptors were determined to be the best combination for reliable and accurate S1R and S2R multiclassification, offering both strong performance and interpretability.

### Regression Models

The two-step-based workflow using regression models included the prediction of pActivity values to identify S1R and S2R active or inactive compounds. The strategy applied in the second step was based on the combination of predicted values to retrieve selective compounds as a difference of pActivity for the two subtypes (Figure 1B). So, the 5 ML algorithms in combination with five features were explored for the development of regression-based QSAR using Data set 1 and Data set 2. The quality of each model was assessed through nested cross-validation, evaluating two key metrics: root mean square error (RMSE) and *R* ^2^. The regression models show reasonable model quality using Data set 1 across outer test folds, especially for XGBoost (RMSE = 0.654 ± 0.014; *R* ^2^ = 0.750 ± 0.008) and RF algorithms (RMSE = 0.678 ± 0.011 and 0.732 ± 0.004) using Mordred descriptors (see Table S7). Unfortunately, the performance declined when applied to Data set 2, which contains fewer data points (Table S8). This outcome highlighted the importance of having enough data to train models effectively and achieve high-quality predictions. Across outer test folds, best quality parameters were accounted for ET (RMSE = 1.149 ± 0.095 and *R* ^2^ = 0.544 ± 0.043) and SVM (RMSE = 1.159 ± 0.104 and *R* ^2^ = 0.535 ± 0.060) models using Mordred descriptors but based on stability criteria ET model was selected as most accurate machine learning model (Table S8). Also, in this case, physical properties defined by Mordred descriptors were demonstrated as the best features to train the models.

Having identified the best model-descriptor combinations via nested cross-validation, we then conducted a classical 5-fold cross-validation using the entire training set to obtain a more stable performance estimate of the final model as no further hyperparameter tuning was required.

#### Classical 5-Fold Cross-Validation and Final Models

After identifying the best algorithm-descriptor combination via nested cross-validation, we retrained each on the entire data set and performed a classical 5-fold cross-validation. In this phase, hyperparameters that consistently showed strong performance during nested cross-validation were held fixed, while any remaining parameters were tuned over a narrower search range. This approach maximizes the use of all available data, avoids redundant tuning, and yields robust models for each corresponding workflow.

### Binary Classification Models

Accordingly, ET and XGBoost were used for S1R, while ET was employed for S2R, all trained on Mordred descriptors. All models were trained on the entire data sets, and the quality parameters derived from the classical 5-fold evaluation are summarized in Table 2.

**Table 2:** Classical 5-Fold Cross-Validation Quality Parameters of Binary Classification Models Selected to Predict Activity^a^

target	model	ROC-AUC	*F*1-score	precision	recall	MCC
S1R	XGBoost: Mordred	0.974 ± 0.004	0.954 ± 0.004	0.975 ± 0.002	0.933 ± 0.008	0.794 ± 0.015
S1R	ET: Mordred	0.972 ± 0.006	0.953 ± 0.005	0.971 ± 0.004	0.936 ± 0.008	0.788 ± 0.020
S2R	ET: Mordred	0.893 ± 0.029	0.88 ± 0.027	0.951 ± 0.010	0.812 ± 0.048	0.545 ± 0.054

a For each metric, the mean value and standard deviation are reported.

As shown in Table 2, the XGBoost and ET models showed very similar performances in predicting S1R activity. Despite the slight difference, quality metrics from the classical 5-fold evaluation confirmed XGBoost as the top-performing model in terms of performance and stability across folds. Regarding S2R activity prediction, the ET model confirmed its performance consistently with nested 5-fold cross-validation (Table 2).

Regarding the selectivity prediction, the quality metrics for the final models are summarized in Table 3. The SVM selectivity models exhibited comparable performance, with an ROC-AUC of approximately 0.87. Slightly higher *F*1-score, precision, and recall values were observed for S1R (≈0.91) compared to S2R selectivity prediction, which ranged between 0.88 and 0.90.

**Table 3:** Classical 5-Fold Cross-Validation Quality Parameters of Binary Classification Models Selected to Predict Selectivity^a^

target	model	ROC-AUC	*F*1-score	precision	recall	MCC
S1R	SVM: ECFP4	0.875 ± 0.031	0.913 ± 0.014	0.918 ± 0.011	0.911 ± 0.018	0.451 ± 0.085
S2R	SVM: ECFP6	0.877 ± 0.046	0.888 ± 0.017	0.901 ± 0.01	0.881 ± 0.022	0.507 ± 0.051

a For each metric, the mean value and standard deviation are reported.

### 3-Multiclass Classification Models

As expected, the larger size of Data set 3 yielded higher quality metrics compared to Data set 4, underlining the crucial role of data size in effectively training ML models. The standard 5-fold cross-validation confirmed previously reported metrics for the selected ET multiclass models. Table 4 presents the performance metrics for the final ET models, evaluated on the classical 5-fold cross-validation test folds.

**Table 4:** Classical 5-Fold Cross-Validation Quality Parameters of 3-Multiclass Classification Models Selected to Predict Activity and Selectivity^a^

target	model	ROC-AUC	*F*1-score	Precision	Recall	MCC
S1R	ET: Mordred	0.94 ± 0.001	0.85 ± 0.018	0.877 ± 0.016	0.84 ± 0.017	0.711 ± 0.023
S2R	ET: Mordred	0.873 ± 0.02	0.762 ± 0.018	0.80 ± 0.021	0.75 ± 0.017	0.723 ± 0.034

a For each metric, the mean value and standard deviation are reported.

It is important to note that S1R-selective and S2R-selective subsets are considerably smaller than other classes, resulting in highly imbalanced data sets. To mitigate overestimation, we examined confusion matrices for each fold (Figures S2 and S3). This approach helps avoid potential misinterpretation of average metrics and provides an opportunity to adjust the models if needed.

These matrices summarize the performance of the ET model across 5-fold classical cross-validation for predicting selectivity and inactivity for S1R and S2R. For both targets, the ET model consistently achieved high accuracy in predicting inactive compounds, as indicated by strong diagonal values across all folds. Nonselective compounds also demonstrated high classification accuracy, though occasional misclassifications with inactive and selective compounds were observed. Predictions for selective compounds were less accurate, with moderate performance and notable misclassifications, probably due to the limited data set representation. Overall, the ET model displayed stable performance across folds for both S1R and S2R, with the most significant challenge being the accurate prediction of selective compounds.

### Regression Models

The selected XGBoost and ET models for S1R and S2R pActivity predictions were submitted to the classical 5-fold cross-validation to obtain the final models, whose results are collected in Table 5. Again, quality metrics for S2R pActivity predictions have underlined that the smaller amount of data negatively affected the performance (Table 5). Despite the limited ability of the ET model to accurately predict pActivity values for S2R compounds, we decided to implement the regression models in Workflow 2 to evaluate their performance on the external validation set.

**Table 5:** Classical 5-Fold Cross-Validation Quality Parameters of Regression Models Selected to Predict pActivity Values^a^

target	model	RMSE	*R* ^2^
S1R	XGBoost: Mordred	0.650 ± 0.016	0.753 ± 0.009
S2R	ET: Mordred	1.146 ± 0.0968	0.545 ± 0.0570

a For each metric, the mean value and standard deviation are reported.

To benchmark the performance of the selected models, we implemented a set of baseline models for both the classification and regression tasks. For selectivity classification, we implemented logistic regression, 1-nearest neighbor, decision tree, and two dummy classifiers representing the majority class prediction and uniform random guessing. To explore potential bias exploitation, we also trained a logistic regression model using only the SLog *P* descriptor. All models were trained and evaluated on Data sets 3 and 4, which report selectivity labels for S1R and S2R, respectively. For the regression workflow, we included a dummy regressor that always predicted the median value of the training set (using Data sets 1 and 2 for S1R and S2R, respectively) as a baseline reference.

Across both tasks, baseline models consistently underperformed relative to our selected models trained on mordred descriptors. Specifically, baseline classifiers yielded lower ROC-AUC scores (ranging from 0.48 to 0.78), *F*1-scores below 0.68, and substantially reduced MCC values (Tables S9 and S10). Likewise, the dummy regressor produced significantly higher RMSE values and near-zero or negative *R* ^2^ scores (Table S11). These findings emphasize the strength of our model selection strategy based on nested cross-validation and confirm that advanced learning algorithms combined with chemically informative descriptors offer clear advantages over simplistic or biased baseline models.

#### External Validation and Workflow Comparison

The external validation set was employed to compare the three proposed workflows and assess their performance and generalizability to unseen data.^37,38^ The external set contained 102 active and 8 active S1R ligands and 101 active and 7 inactive S2R compounds. From a selectivity perspective, it included 88 nonselective, 8 S1R-selective, 12 S2R-selective, and 4 nonbinders. The external validation set provides an accurate representation of the distribution of selective and nonselective compounds for SRs, enabling it to be a valuable tool for validation. The limited number of molecules in the data set may significantly impact model performance across different workflows, potentially introducing variability. We recognized that this small sample size restricted the generalizability of our conclusions. Nonetheless, to reinforce the robustness of our findings, we incorporated an external validation step, underscoring our commitment to an in-depth analysis despite this limitation. Our final goal was to predict selectivity, which is fundamentally a classification task (e.g., in the 2-step regression workflow, molecules are categorized according to a predefined threshold). Hence, a realistic estimation of the predictive performance of the models can be provided by judging the model performance based on classification validation metrics. To compare the three workflows, ROC-AUC and MCC score and classification reports providing detailed metrics for each label, including precision, recall, *F*1-score, and support (i.e., number of compounds for each class), were employed. Additionally, confidence intervals (CIs) for these metrics were reported. In general, CIs obtained for selective classes prediction (Tables 6 and 8) indicate moderate uncertainty, which is primarily due to the low prevalence of selective compounds, especially in the case of S1R. In this study, we implemented and evaluated workflows based on their ability to identify selective compounds.

**Table 6:** Classification Report of the SVM Binary Classification Model for the S1R Selectivity Prediction

class	*F*1-score (95% CI)	precision (95% CI)	recall (95% CI)
nonselective	0.95 (0.92–0.98)	0.97 (0.93–1)	0.94 (0.91–0.97)
S1R-selective	0.18 (0.080–0.274)	0.14 (0.035–0.235)	0.25 (0.152–0.352)

### 2-Step Classification Workflow

In summary, the 2-step classification workflow operates in two stages. In the first step, XGBoost (using Mordred descriptors) and ET (using Mordred descriptors) models were applied to predict the activity for S1R and S2R, respectively. After combining the results, only compounds predicted as active were advanced to the selectivity prediction stage, which used SVM with ECFP4 fingerprints for S1R and SVM with ECFP6 fingerprints for S2R. From the first step, 100 compounds were predicted to be active for both S1R and S2R. In the second step, the SVM model with ECFP4 fingerprints identified 90 nonselective compounds, labeling 6 compounds as selective. However, among the remaining four compounds, only one was correctly predicted as selective, resulting in the metrics reported in Table 6. Notably, the overall performance of the model provided a ROC-AUC = 0.594 (CI = 0.49–0.68) and MCC = 0.144 (CI = 0.05–0.44).

For S2R, the SVM model with ECFP6 fingerprints performed better, correctly identifying 84 nonselective compounds out of 90 and 7 selective compounds out of 10 (Table 7). This performance resulted in the following metrics: ROC-AUC = 0.817 (CI = 0.720–0.9) and MCC = 0.565 (CI = 0.47–0.66).

**Table 7:** Classification Report of the SVM Binary Classification Model for the S2R Selectivity Prediction

class	*F*1-score (95% CI)	precision (95% CI)	recall (95% CI)
nonselective	0.95 (0.92–0.98)	0.95 (0.93–0.97)	0.93 (0.89–0.97)
S2R-selective	0.61 (0.34–0.72)	0.54 (0.37–0.83)	0.70 (0.47–0.89)

### 2-Step Regression Workflow

In the 2-step regression workflow, XGBoost and ET algorithms, utilizing Mordred descriptors, were employed to predict the pActivity values of compounds for S1R and S2R. The data sets were merged, and selectivity was calculated as the difference between the predicted pActivity values for each compound. Compounds with pActivity >5 and a difference ≥2 (corresponding to a 100-fold selectivity) were labeled as selective, while those with pActivity >5 and a difference <2 were categorized as nonselective. As expected, this approach failed to accurately predict selectivity, achieving a ROC-AUC of only 0.78 (CI = 0.7–0.86). The results revealed that only nonselective compounds were correctly classified, with no accurate predictions for selective compounds (Table 8). The poor performance may be attributed to the lower accuracy of the ET model in predicting selectivity for S2R compared with the XGBoost model used for S1R. Moreover, as previously noted, the quantity of training data is crucial for building high-quality ML models. In this case, the imbalance in data points between the two SR subtypes, particularly for regression analysis, significantly contributed to the failure of this approach.

**Table 8:** Classification Report of the ET Regression Models for SRs Selectivity Prediction

class	*F*1-score (95% CI)	precision (95% CI)	recall (95% CI)
nonselective	0.89 (0.84–0.93)	0.82 (0.74–0.89)	0.98 (0.94–1)
S1R-selective	0.00	0.00	0.00
S2R-selective	0.00	0.00	0.00

### 1-Step Multiclass Workflow

Interestingly, these models achieved high performance in predicting the three classes for both sigma subtypes. Regarding S1R prediction, an overall accuracy of 0.936 (CI = 0.88–0.97) and an MCC of 0.733 (CI = 0.56–0.88) were reported. The model correctly predicted 94 out of 95 nonselective compounds, 5 out of 7 inactives, and 4 out of 8 selective compounds, misclassifying 3 as inactives and 1 as nonselective (Table 9). Table 9 Report for S1R activity and selectivity using ET multiclass classification models.

**Table 9:** Classification Report of the ET Multiclassification Model for S1R Activity and Selectivity Prediction

class	*F*1-score (95% CI)	precision (95% CI)	recall (95% CI)
S1R-selective	0.6 (0.4 −0.86)	0.62 (0.3–1)	0.5 (0.2–0.86)
nonselective	0.984 (0.96–1)	0.98 (0.95–1)	0.99 (0.97–1)
inactive	0.571 (0.33–0.89)	0.67 (0.29–1)	0.7 (0.3–1)

When evaluated on the external set, the ET model trained with Data set 6 (encompassing S2R activity and selectivity definitions) achieved an overall ROC-AUC of 0.964 (CI = 0.93–0.99) and an MCC of 0.89 (CI = 0.77–0.98), making it the most predictive model for S2R. It predicted 87 nonselective compounds out of 90 true ones, correctly classified all inactive compounds, and correctly labeled 11 out of 12 as S2R-selective compounds (Table 10).

**Table 10:** Classification Report of the ET Multiclassification Model for S2R Activity and Selectivity Prediction

class	*F*1-score (95% CI)	precision (95% CI)	recall (95% CI)
S2R-selective	0.88 (0.71–1)	0.846 (0.62–1)	0.917 (0.73–1)
nonselective	0.977 (0.95–0.99)	0.988 (0.96–1)	0.967 (0.93–1)
inactive	0.941 (0.8–1)	0.889 (0.66–1)	1.00 (1–1)

Nevertheless, it is important to note that the conclusions drawn from these results are limited by the small external validation set size, which makes it challenging to ensure robustness of the predictions, especially for S1R-selective compounds. Larger validation sets would be necessary to confirm the observed trends and model performance more reliably.

#### Feature Importance Analysis and Correlation with Selective Compounds

To gain mechanistic insights into the predictive models and enhance interpretability, we evaluated the feature importance scores derived from the ET multiclass models trained on Data sets 5 and 6 (Figure 4A,4B, respectively). A description of each feature is provided in Table 11, based on the official Mordred documentation (https://mordred-descriptor.github.io/documentation/master/descriptors.html) and literature^39^

**Figure 4:**
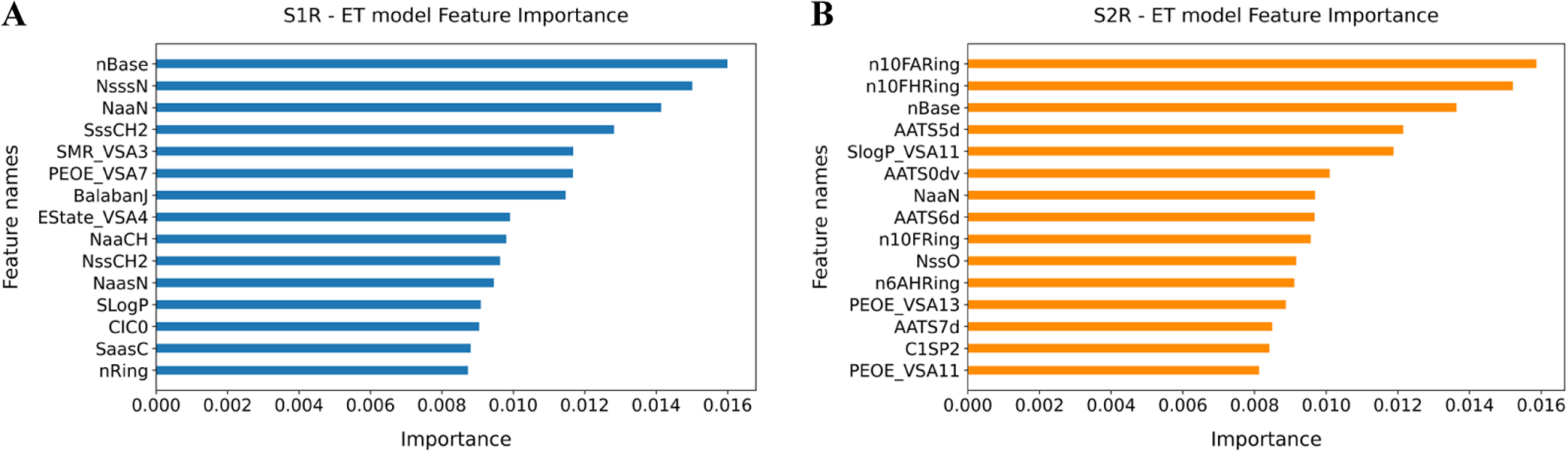
Feature importance derived from the ET models using Mordred descriptors for S1R-selective (A) and S2R-selective (B) compounds.

**Table 11:** List of Mordred Molecular Descriptors Used for Model Interpretation^a^

descriptor	description
nBase	count of basic groups (typically atoms with available lone pairs, like amines)
NsssN	number of nitrogen atoms (N) bonded to three single-bonded atoms (e.g., trialkylamine-type)
NaaN	number of nitrogen atoms bonded to two aromatic atoms (aryl–*N*-aryl connectivity)
SssCH2	sum of methylene (CH_2_) groups bonded to three single-bonded atoms (branched CH_2_ centers)
SMR_VSA3	MOE-type van der Waals surface area (VSA) contribution binned by molar refractivity (bin 3)
PEOE_VSA7	VSA descriptor binned by partial charge (PEOE)—bin 7 (specific charge range)
Balaban J	Balaban J index (a topological descriptor reflecting molecular complexity and connectivity)
EState_VSA4	sum of E-state indices over atoms contributing to VSA bin 4 (approximately 0.72 ≤ *x* < 1.17)
NaaCH	number of methine (CH) groups bonded to two aromatic atoms (aryl–CH–aryl)
NssCH2	number of methylene (CH_2_) groups bonded to two single-bonded atoms
NaasN	number of nitrogen atoms bonded to two aromatic and one single-bonded atom
SLog *P*	Wildman–Crippen calculated Log *P* (hydrophobicity estimation)
SLog *P*_VSA11	VSA contribution to Log *P* from surface area bin 11 (highly lipophilic surface)
CIC0, CIC1	complementary information content indices of orders 0 and 1 (graph-based molecular complexity)
SaasC	number of carbon atoms bonded to two aromatic and one single-bonded atom
nRing	total number of rings in the molecule
TopoPSA(NO)	topological polar surface area calculated from nitrogen and oxygen atoms
PEOE_VSA11, PEOE_VSA13	VSA contributions in charge bins 11 and 13 (partial charge-based surface contributions)
AATS 5d	Broto–Moreau autocorrelation of lag 5, weighted by σ electrons
AATS0dv	Broto–Moreau autocorrelation of lag 0, weighted by valence electrons
AATS6d, AATS7d	same as above, computed with lags of 6 and 7
AATSC0v	centered autocorrelation of lag 0, weighted by valence electrons
n10FARing	number of 10-membered fluorinated aromatic rings
n10FHRing	number of 10-membered fluorinated heteroaromatic rings
n10FRing	number of 10-membered rings containing at least one fluorine atom
n6AHRing	number of 6-membered aromatic heterocycles
C1SP2	number of carbon atoms with sp^2^ hybridization and only one connection (typically terminal =CH groups)
IC1	information content index of order 1 (measures graph-based structural complexity)

a Each descriptor is reported along with its name and a brief description.

For the S1R-selective model, the most influential descriptors included nBase, NsssN, and NaaN, highlighting the importance of basic nitrogen atoms and their connectivity, particularly to tertiary and aromatic environments. These findings are consistent with the established pharmacophore and structural models of S1R ligands, which emphasize a central basic amine flanked by two hydrophobic regions, crucial for interacting with the conserved Glu172 residue within the S1R binding pocket.^40–43^ Additional top-ranked descriptors, such as SMR_VSA3 and PEOE_VSA7, reflect the molecular polarizability and partial atomic charges, respectively. While not specific to basic amines, these features further support the role of electrostatic complementarity and polar surface interactions in high-affinity S1R binding (Figure 4A).

In contrast, the S2R-selective model prioritized descriptors such as n10FARing, n10FHRing, and hydrophobic features, including AATS 5d and SLog *P*_VSA11, suggesting that fluorinated aromatic ring substitution and overall lipophilicity are key determinants of S2R selectivity. While nBase remained among the top-ranked features, the absence of NaaN supports the idea that basicity is generally important for SRs binding (i.e., activity) but that its role in selectivity is context-dependent and mediated by distinct structural frameworks. These findings are supported by previous observations that S2R ligands often exhibit bulkier, halogen-rich aromatic scaffolds, favoring hydrophobic interactions within the S2R binding site (Figure 4B).^44–49^

To better understand the feature profiles of highly selective compounds, we examined the top-scoring molecules (Figure 5) for each subtype, based on the prediction of the ET models (Tables S12 and S13).

**Figure 5:**
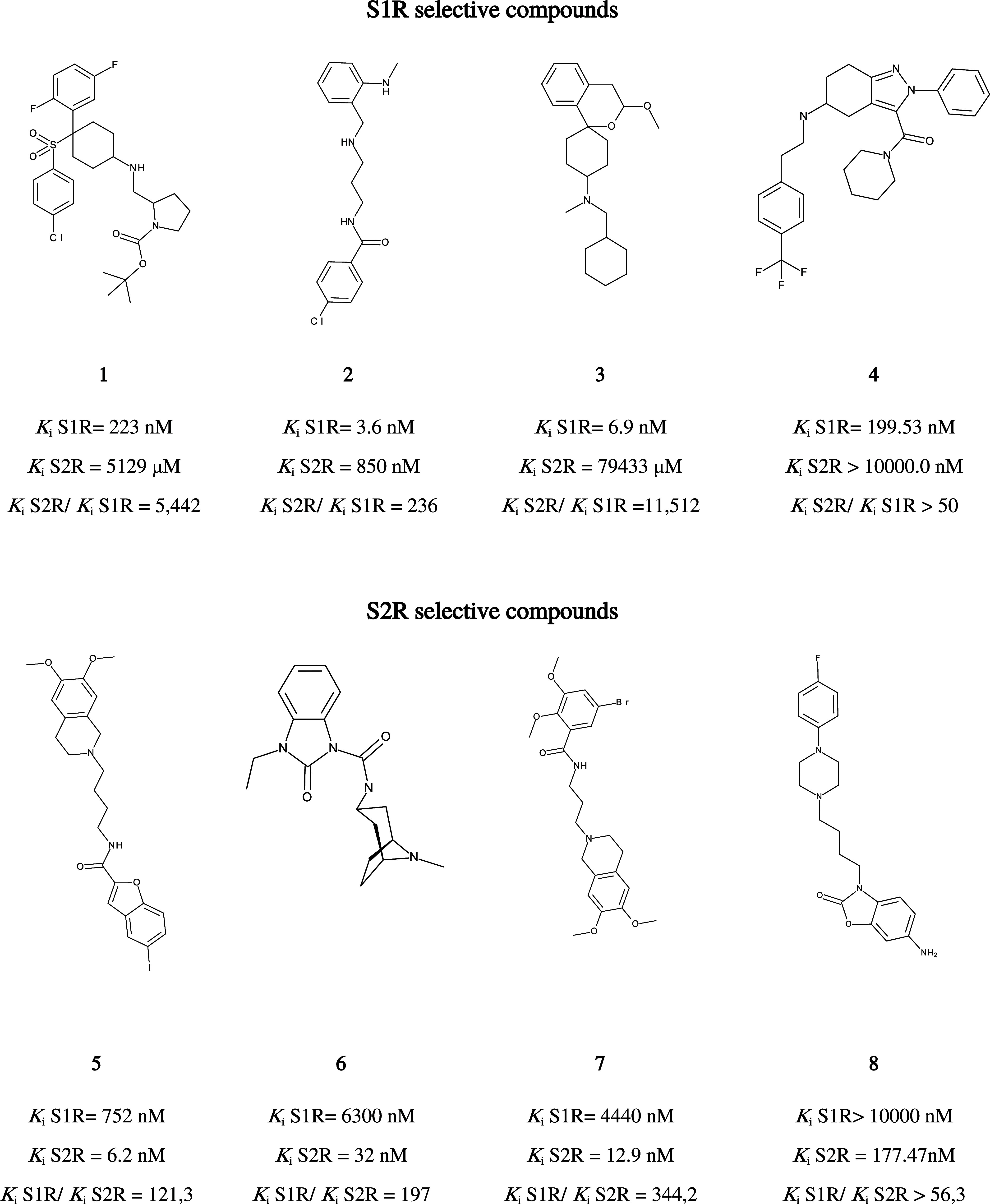
Chemical structures of top-ranked selective ligands as predicted by the model, along with their experimental *K*_i_ values and selectivity ratios.

In accordance with the structural requirements for binding SRs, including the presence of basic nitrogen, all selective ligands analyzed share a basic center (nBase = 1). However, the selectivity arises from distinct physicochemical signatures. S1R-selective compounds are characterized by nitrogen-centered topological features (NsssN and NaaN), with high selectivity achieved through varied combinations of molecular rigidity, hydrophobicity, and electrostatics. For instance, compound **3** showed exceptional S1R selectivity (S_2_R/S_1_R > 11,000), driven by a simple scaffold and high SssCH2, whereas compound **4**, with elevated values of EState_VSA4, Balaban J, and nRing, suggests that topological complexity and electronic distribution can also drive selectivity (S_2_R/S_1_R > 50). In contrast, S2R-selective ligands lacked NaaN and displayed consistently high values for lipophilic surface area descriptors such as SLog *P*_VSA11 and PEOE_VSA11, as well as halogenated ring counts (n10FARing, n10FHRing, and n10FRing). Highly S2R-selective ligands like compounds **5** and **7** exhibited these features prominently. Collected findings underscore that while basicity is a shared requirement, S1R selectivity is more dependent on electronic and topological patterns, whereas S2R ligands rely on hydrophobicity, bulk, and halogenated aromatic substitution for subtype preference. Table 12 reports information associated with each selectivity class, highlighting differences in the nitrogen content, hydrophobicity, aromaticity, surface area, and molecular complexity.

**Table 12:** Overview of the Descriptor Profiles Distinguishing S1R- and S2R-Selective Ligands^a^

descriptor type	S1R-selective compounds	S2R-selective compounds
basic nitrogens	high values for NsssN, NaaN, nBase (always 1)	nBase = 1 in all compounds; absence of NaaN
hydrophobicity	moderate SLog *P*; not a dominant feature	high SLog *P*_VSA11, indicating strong lipophilicity
aromatic rings	moderate ring counts (nRing)	presence of fluorinated aromatic rings: n10FRing, n10FARing, n10FHRing
van der Waals surface	high PEOE_VSA7, SMR_VSA3	high PEOE_VSA13, PEOE_VSA11
topological complexity	Balaban J, CIC0, EState_VSA4	AATS0dv, AATS 5d, AATS6d, C1SP2 prominent
selectivity insights	driven by electrostatics and basic centers	driven by surface area, bulk, and hydrophobicity

a The table summarizes key physicochemical and topological features related to each selectivity class.

## Conclusions

In this study, three distinct workflows were developed using various ML approaches, including binary classification, multiclass classification, and regression. A combination of 5 ML algorithms (RF, ET, XGBoost, SVM, and *k*NN) paired with five molecular representations (RDKit descriptors, Mordred descriptors, ECFP4, ECFP6, and MACCS) led to the creation of 200 unique ML models. Each model was developed using six distinct Data sets, carefully tailored to maximize model efficiency. However, this approach posed challenges for directly comparing the performance of different workflows, as the models were trained and tested on varied data sets. To address this, an external validation set was employed to provide a consistent benchmark. The limited number of molecules in the data set, particularly for selective S1R and S2R compounds, could significantly impact model performance across workflows, introducing potential variability. We acknowledge that this small sample size constrains the generalizability of our conclusions. Nevertheless, to enhance the robustness of our findings, we incorporated an external validation step, demonstrating our commitment to conducting a comprehensive and rigorous analysis despite these limitations. We found that the 1-step multiclass workflow showed the strongest overall results for predicting SR activity and selectivity in a single model. Tree-based ensemble methods, such as XGBoost and ET, demonstrated superior efficiency. The comparable performance of XGBoost and ET underscores the suitability of Mordred descriptors for this type of prediction and highlights the advantages of ensemble-based methods in achieving high accuracy and balanced predictive capabilities. In the 2-step regression workflow, a key limitation in training regression models for SRs is the scarcity of available data, particularly for S2R, which comprises only 25% of the data set available for S1R. This data imbalance significantly impacted the regression model performance, especially for S2R. While the 2-step classification workflow also proved effective during the training and testing phases, external validation revealed challenges in accurately classifying S1R-selective compounds. Nevertheless, promising results were achieved for S2R-selective compounds. It is important to note that the relatively small size of the external validation data set, particularly for S1R and S2R selectivity, may have influenced model performance. Despite these challenges, our findings lay the groundwork for a time-efficient and accurate method to identify active and selective compounds for SRs. In addition, feature importance analysis and investigation of top-ranked model predictions revealed clear trends underlying the S1R and S2R selectivity. S1R-selective ligands typically exhibited enhanced basicity and lower lipophilicity, while S2R-selective compounds were associated with an increased hydrophobic surface area and extensive aromatic substitution. This approach aims to enhance predictive models’ overall reliability and applicability in drug discovery workflows.

## Materials and Methods

### Data Set Preparation

The initial data sets for S1R and S2R were extracted from different public sources: ChEMBL release 34,^27^ PubChem BioAssays,^29^ BindingDB,^28^ and S2RSLDB,^30^ using the UniProt ID Q99720 for S1R and Q5BJF2 for S2R (accessed on April 26th, 2024) along with in-house compounds.^31,32^ For both receptors, data collected from different sources were merged and then filtered to retain structures with biological activities expressed as *K*_i_, IC_50_, *K*_d_, EC_50_ with a standard relation set as “=” or “>” when activity was higher than 10,000 nM using python library Pandas version 2.2.2.^50^ Data sets were preprocessed by canonizing the SMILES, removing the duplicates, stripping salts, and without changing the molecules. All of these steps were performed using RDKit version 2024.3.5 (RDKit: Open-source cheminformatics, https://www.rdkit.org).^51^ As for some compounds, several activity records were reported, and a procedure with the following criteria was applied. First, duplicated compounds with standard relation set to “=” were prioritized over compounds with standard relation recorded as “>”. For compounds with activity measurements reported using the “>” qualifier, the highest value was retained. This approach reflects the maximum concentration tested in biological assays and ensures a conservative interpretation of inactivity. Then, *K*_i_ values were prioritized over IC_50_ and *K*_d_ recorded data. For the remaining multiple compounds, the mean value was calculated. Molecules were retained if the standard deviation was less than 20% of the original mean value. Conversely, compounds were discarded if the standard deviation exceeded the threshold. In the end, activity values were converted into pActivity (the negative logarithmic value of the molar activity concentration) to standardize the different measures following the pChEMBL convention.^52^ The applied procedure resulted in an S1R/S2R data set comprising 3616 compounds from ChEMBL, 286 from PubChem, 1046 from S2RSLDB, 908 from BindingDB, and 34 from an in-house database. In total, the data set included 4552 activity data points for S1R and 1490 activity data points for S2R. Compounds in the S1R/S2R were defined based on activity and selectivity criteria. Initially, compounds with activity records for both receptors were labeled as “double points”, whereas “single points*”* referred to compounds with biological data for only one of the two receptors. Compounds were further labeled as actives if pActivity >5 and inactives with a pActivity ≤5. For double points, i.e., compounds with bioactivity data for both SR subtypes, selectivity categories were also added based on the following conditions. The molecules were labeled as “S1R-selective” if pActivity >5 for S1R and activity greater than 100-fold (pActivity >2) when compared with S2R value or if the corresponding S2R pActivity was ≤5 (i.e., classified as inactive). On the other hand, molecules were classified as “S2R-selective” when pActivity >5 for S2R and activity greater than 100-fold (pActivity >2) when compared with S1R data, or if the corresponding S1R pActivity was ≤5 (i.e., classified as inactive). Compounds inactive against both targets (pActivity ≤5) were labeled as “nonbinders”. The remaining compounds with pActivity >5 and difference in activity ≤100-fold were assigned to the “nonselective” category. For the S1R data set, compounds that were selective for S2R were labeled as “nonselective” and vice versa. Therefore, from the original data set, we extracted 10% of double points (S1R-selective, S2R-selective, nonselective, and nonbinders) to ensure a fixed external data set for consistent evaluation of the workflow bases in binary classification, multiclass classification, and regression ML models. The remaining compounds were used to generate different data sets based on classification and regression tasks, obtaining six data sets. The maximum Tanimoto similarity was calculated for each compound in the external validation set with respect to the double-point compounds in the training set. Similarity was computed using ECFP4 fingerprints, and the Tanimoto coefficient of the RDKit library^51^ was used as the similarity metric. The same approach was applied to evaluate intradata set chemical similarity across the six data sets (Data sets 1–6) used to train the different ML models.

#### t-Distributed Stochastic Neighbor Embedding (t-SNE)

The chemical similarity between the training set (including only double-point compounds) and the external validation set was assessed using t-SNE.^53^ Compounds were encoded as ECFP4 fingerprints, and dimensionality reduction was performed using the TSNE function from scikit-learn with the following parameters: *n*_components = 2, perplexity = 30, and learning_rate = 10. The resulting t-SNE dimensions 1 and 2 were used for visualization.

### Molecular Representation and Feature Selection

To develop ML models that are able to distinguish between active and inactive, as well as selective and nonselective SRs molecules, the use of molecular descriptors or fingerprints is required. Therefore, each compound was described using different molecular representations. MACCS keys fingerprints,[Bibr ref26] 2048-bit Morgan fingerprints with radius = 2 (roughly equivalent to ECFP4), and Morgan fingerprints with radius = 3 (corresponding to ECFP6)^25^ were calculated by RDKit. Additionally, molecular descriptors were calculated using Mordred^23^ for 1613 2D molecular descriptors and RDKit,^54^ resulting in 125 molecular descriptors. Hence, a procedure to reduce the number of Mordred and RDKit descriptors was implemented. In the first step, descriptors with missing values were removed. Therefore, low-variance descriptors were removed using the cutoff value 0.1. In the last step, a correlation matrix was evaluated as implemented in Python to retain only nonredundant features that provided a Pearson correlation coefficient (PCC) lower than 0.9. In total, 5 arrays of molecular representations were generated for modeling testing and to investigate a better representation in recognizing molecules.

### Machine Learning Algorithms and Evaluation

All metrics were used to evaluate the model developed by using different molecular descriptors and fingerprints. Based on the overall performance and stability of the models, the best models were selected to carry out a classical 5-fold cross-validation to train and test models again using the entire data set. In this study, 5 ML algorithms were utilized to build models for the regression and classification tasks: random forest (RF),^55^ extreme gradient boosting (XGBoost),^56^ support vector machines (SVMs),^57^ *k*-nearest neighbors (*k*NN),^58,59^ and extra trees (ET).^59^ The scikit-learn module^60^ (version 1.4.2) and the XGBoost Python library (version 2.1.1).^56^ were used to implement these models. For classification tasks, compounds were labeled as “1” (active) and “0” (inactive). For multiclassification tasks, compounds were categorized as “1” (selective), “2” (nonselective), and “0” (inactive). For the regression tasks, pActivity was used as the target variable. To evaluate the generalization performance of the models on unseen data, a nested cross-validation procedure was applied, splitting each data set into 5 outer folds. The outer loop was used to evaluate model performance on unseen data, while the inner loop was dedicated to hyperparameter tuning. In each outer loop iteration, 80% of the data was used for training and 20% was held out for testing. To handle class imbalance in classification and multiclassification models, the RandomUnderSampler method from the imbalanced-learn module was applied to balance the training data by randomly selecting subsets of data for targeted classes. Undersampling was preferred over oversampling to avoid synthetic data generation artifacts and to preserve the chemical space integrity of the minority class. Additionally, to address the class imbalance, several strategies were applied across different models. For the RF, SVM, and ET models, class weights were adjusted using the “class_weight” parameter to balance the influence of different classes during training. For XGBoost, the “sample weight” method was used to assign varying weights to individual samples, providing greater flexibility in emphasizing underrepresented samples. In the *k*NN model, the distance metric was configured to weigh the influence of each neighbor by its proximity, ensuring that closer neighbors have a more significant impact; it is an approach particularly useful for imbalanced data sets. For classification tasks, Stratified *k*-fold cross-validation was employed to preserve class distribution within each fold. For regression tasks, *k*-fold cross-validation was used. In the inner loop of the nested cross-validation, 5-fold cross-validation was performed to tune model hyperparameters. In both methods, the default behavior divides the data set into *k* equal-sized parts, where one part is used as the test set (20% of the data for 5-fold), and the remaining *k* – 1 parts (80% of the data for 5-fold) are used as the training set. Stratified *k*-fold ensures that the class distribution in the target variable (or proportions of the target variable’s categories) is preserved in each fold, which is especially useful for data sets with imbalanced target classes. Hyperparameter optimization was conducted using the Optuna framework^61^ with the tree-structured Parzen estimator (TPE) sampler, performing 50 trials per iteration. In the inner loop of the nested cross-validation, hyperparameters were initially tuned across the ranges and options listed in Table 11. Those parameters that consistently yielded strong performance were held fixed during the subsequent classical 5-fold cross-validation, while any remaining hyperparameters were tuned over a narrower range (Table 13).

**Table 13:** Hyperparameters Search Space for Each ML Model

ML algorithms	hyperparameters
ET	bootstrap = [true, false], max_features = [“sqrt”,“log 2”], *n*_estimators = (100, 1000), max_depth = (3, 30), min_samples_split = (2, 20), min_samples_leaf = (1, 10), criterion = [“squared_error”, “absolute_error”, “friedman_mse”, “poisson”], class_weight = [“balanced”, “balanced_subsample”]
RF	bootstrap = [true,“log 2”, false], max_features = [“sqrt”], *n*_estimators = (100, 1500), max_depth = (5, 80), min_samples_split = (2, 20), min_samples_leaf = (1, 6), criterion = [“squared_error”, “absolute_error”, “friedman_mse”, “poisson”], class_weight = [“balanced”, “balanced_subsample”]
XGBoost	*n*_estimators = (10, 1000), max_depth = (3, 30), learning_rate = (0.01, 0.3), subsample = (0.5, 1), colsample_bytree = (0.5, 1), sample_weights = class_weight = [“balanced”, “balanced_subsample”]
SVM	*C* = (0.01, 100.0), γ = (1^e–4^, 1.0), ε = (1, 1.0), class_weight = balanced^a^
*k*NN	*n*_neighbors = (2, 30), weights = [“distance”,^a^ “uniform”], metric = [“minkowski”, “euclidean”, “manhattan”], algorithm = [“auto”, “ball_tree”, “kd_tree”, “brute”], leaf_size = (1, 50)

a Parameters set for binary and multiclassification ML models.

The ROC-AUC^62^ was used as the optimization metric for binary and multiclass classification models, while root mean square error^63^ (RMSE) was the optimization metric for regression models. Model evaluation included statistical metrics such as ROC-AUC, *F*1-score, precision, recall,^64^ and Matthew’s correlation coefficient^65^ (MCC) for binary and multiclassification classification tasks.

For both binary and multiclass classification models, the score was calculated using a weighted average to account for class imbalance, while the one-vs-rest (OvR) strategy was used for multiclass scenarios.

root mean square error (RMSE) and the coefficient of determination (*R* ^2^) were used to evaluate the regression models’ performance. The final metrics were averaged across the outer folds to ensure robust and unbiased assessments.

Hence, based on the overall performance and stability, the best-performing models were selected to undergo an additional 5-fold cross-validation using the entire data set for further training and testing. The joblib module^66^ in Python was utilized to save and load selected features and models for the workflows, ensuring efficient reuse and reproducibility. Additionally, the classification_report module from scikit-learn was employed to generate detailed metrics for each class, facilitating the evaluation of the model performance during external validation and workflow comparison.

#### Baseline Model Implementation

To contextualize the performance of our selected models, we compared them with a range of baseline models. For classification, the following models were evaluated: logistic regression,^67^ 1-nearest neighbor classifier, decision tree classifier,^68^ logistic regression on SLog *P* only, dummy classifiers (https://scikit-learn.org/stable/modules/generated/sklearn.dummy.DummyClassifier.html) using most frequent (always predicts the majority class), and uniform (randomly predicts class labels) methods. For regression tasks (i.e., pActivity prediction), a Dummy Regressor (https://scikit-learn.org/stable/modules/generated/sklearn.dummy.DummyRegressor.html) was implemented that always predicts the median pActivity value of the training data. All models were evaluated using 5-fold stratified cross-validation (for classification) or standard 5-fold cross-validation (for regression). As for ML models investigated, the same classification (ROC-AUC, *F*1-score, precision, recall, and MCC) and regression (RMSE and *R* ^2^ score) metrics were computed. All models were implemented using scikit-learn (version 1.4.2).

### Feature Importance Analysis

To identify the most influential molecular descriptors contributing to subtype classification, feature importance analysis was performed using the trained extra trees (ET) classifiers for S1R and S2R prediction. The models, embedded within scikit-learn pipelines, were reloaded using the joblib library along with the corresponding subsets of preselected features. Feature importances were retrieved from the “feature_importances” attribute of the fitted ExtraTreesClassifier, and the top 15 most relevant features were selected for further analysis.

## Supplementary Material





## Data Availability

Python codes to generate data sets and to train and test ML models, as well as original databases and curated ones, can be downloaded from the GitHub repository: https://github.com/lisa-lombardo/SRs_ML-based_workflows.git.

## Notes

Comprehensive list of all compounds, including canonical SMILES, associated activity data, and their inclusion in Data sets 1–6 (XLSX)
Tables of nested 5-fold cross-validation quality metrics of binary classification models evaluated for the 2-step classification workflow; tables of nested 5-fold cross-validation quality metrics for multiclass classification + models evaluated for the 1-step multiclassification workflow; tables of nested 5-fold cross-validation quality metrics for regression models evaluated for the 2-step regression workflow; and confusion matrices evaluating the performance of the extra trees multiclassification models across 5-fold classical cross-validation in predicting activity and selectivity profiles for S1R and S2R (PDF)

## Abbreviations

ECFP: extended connectivity fingerprint
ET: extra trees
EC_50_: half-maximal effective concentration
FN: false negative
FP: false positive
IC_50_: half-maximal inhibitory concentration
*K*_d_: dissociation constant
*K*i: inhibition constant
*k*NN: *k*-nearest neighbors
ML: machine learning
nM: nanomolar
PCC: pearson correlation coefficient
QSAR: quantitative structure–activity relationship
RF: random forest
RMSE: root mean error
ROC-AUC: area under the receiver operating characteristic curve
S1R: sigma 1 receptor
S2R: sigma 2 receptor
SRs: sigma receptors
SVM: support vector machine
Tc: tanimoto coefficient
t-SNE: t-distributed stochastic neighbor embedding
TP: true positive
TR: true negative
XGBoost: extreme gradient boosting
