# Supplementary material for: Prediction of Activity and Selectivity Profiles of Sigma Receptor Ligands Using Machine Learning Approaches
Source: J Chem Inf Model. 2025 Sep 1;65(18):9697–712. doi: 10.1021/acs.jcim.5c01091 (PMC12458689; doi:10.1021/acs.jcim.5c01091)
Supplement: Supplementary file 2 [file ci5c01091_si_002.pdf]

Supporting Information

Prediction of Activity and Selectivity Profiles of Sigma Receptor Ligands Using Machine Learning Approaches

Lisa Lombardo,[a] *Verena Battisti,*[b] *Thierry Langer,*[b] *Rosaria Gitto,*[a] *and Laura De Luca*[a]

[a]CHIBIOFARAM Department University of Messina, I-98166 Messina, Italy

[b] Department of Pharmaceutical Chemistry, University of Vienna, Althanstraße 14, A-1090 Vienna, Austria

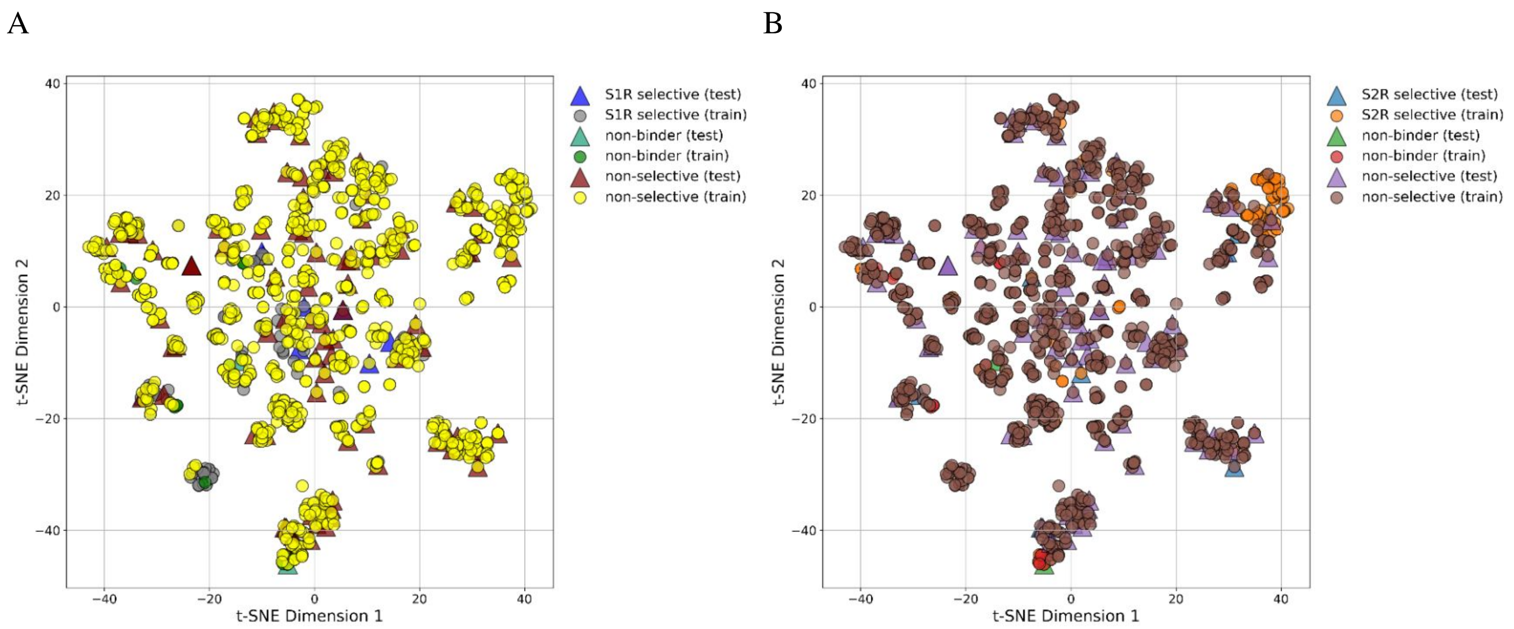

Figure S1. t-SNE visualization of chemical similarity based on ECFP4 fingerprints for compounds in the training set (restricted to double points) and the external validation set. (A) S1R-related selectivity classes: S1R-selective, non-selective, and non-binder. (B) S2R-related selectivity classes: S2R-selective, non-selective, and non-binder. Each point represents a compound, with shape distinguishing training vs test set origin and color indicating selectivity class.

Tables of nested 5-fold cross-validation quality metrics of binary classification models evaluated for the two-step classification workflow.

Table S1A. Nested 5-fold cross-validation quality parameters of binary classification models predicting S1R active and inactive classes. For each metric, the mean value and standard deviation are reported.

Model	Descriptor	ROC_AUC	F1-score	Precision	Recall	MCC
XGBoost	rdkit	0.966 ± 0.002	0.944 ± 0.007	0.972 ± 0.006	0.917 ± 0.014	0.757 ± 0.027
	mordred	0.973 ± 0.006	0.954 ± 0.006	0.975 ± 0.004	0.933 ± 0.009	0.794 ± 0.024
	ECFP4	0.964 ± 0.007	0.943 ± 0.008	0.974 ± 0.006	0.914 ± 0.012	0.757 ± 0.029
	ECFP6	0.963 ± 0.007	0.940 ± 0.007	0.974 ± 0.007	0.909 ± 0.011	0.749 ± 0.026
	MACCS	0.957 ± 0.009	0.944 ± 0.010	0.966 ± 0.005	0.924 ± 0.017	0.754 ± 0.037
ET	rdkit	0.965 ± 0.004	0.944 ± 0.007	0.968 ± 0.002	0.922 ± 0.014	0.755 ± 0.022
	mordred	0.971 ± 0.007	0.954 ± 0.005	0.971 ± 0.005	0.937 ± 0.009	0.790 ± 0.023
	ECFP4	0.968 ± 0.007	0.946 ± 0.007	0.976 ± 0.005	0.917 ± 0.010	0.768 ± 0.028
	ECFP6	0.967 ± 0.006	0.942 ± 0.004	0.976 ± 0.005	0.910 ± 0.003	0.756 ± 0.018
	MACCS	0.958 ± 0.011	0.947 ± 0.006	0.963 ± 0.008	0.931 ± 0.008	0.757 ± 0.030
KNN	rdkit	0.952 ± 0.009	0.955 ± 0.008	0.952 ± 0.006	0.932± 0.011	0.775 ± 0.036
	mordred	0.956 ± 0.010	0.954 ± 0.009	0.952 ± 0.014	0.940 ± 0.006	0.778 ± 0.051
	ECFP4	0.951 ± 0.006	0.729 ± 0.065	0.986 ± 0.004	0.583 ± 0.086	0.446 ± 0.062
	ECFP6	0.930 ± 0.007	0.189 ± 0.073	0.990 ± 0.009	0.106 ± 0.046	0.144 ± 0.032
	MACCS	0.943 ± 0.011	0.954 ± 0.005	0.951 ± 0.006	0.959 ± 0.005	0.772 ± 0.026
RF	rdkit	0.965 ± 0.004	0.940 ± 0.009	0.970 ± 0.006	0.912 ± 0.017	0.744 ± 0.032
	mordred	0.972 ± 0.005	0.949 ± 0.005	0.976 ± 0.004	0.923 ± 0.007	0.777 ± 0.018
	ECFP4	0.970 ± 0.007	0.950 ± 0.006	0.975 ± 0.004	0.926 ± 0.008	0.781 ± 0.025
	ECFP6	0.967 ± 0.006	0.940 ± 0.006	0.975 ± 0.004	0.908 ± 0.007	0.749 ± 0.021
	MACCS	0.959 ± 0.011	0.946 ± 0.005	0.964 ± 0.009	0.930 ± 0.003	0.755 ± 0.029
SVM	rdkit	0.956 ± 0.008	0.945 ± 0.006	0.969 ± 0.003	0.924 ± 0.013	0.759 ± 0.022
	mordred	0.966 ± 0.008	0.949 ± 0.005	0.973 ± 0.005	0.926 ± 0.009	0.776 ± 0.020
	ECFP4	0.961 ± 0.008	0.933 ± 0.009	0.973 ± 0.005	0.896 ± 0.013	0.727 ± 0.031
	ECFP6	0.961 ± 0.008	0.927 ± 0.007	0.976 ± 0.008	0.883 ± 0.012	0.716 ± 0.027
	MACCS	0.952 ± 0.008	0.943 ± 0.009	0.965 ± 0.007	0.921 ± 0.020	0.747 ± 0.026

Table S1B. ROC_AUC values of XGBoost and ET models across the five outer folds of nested cross-validation for S1R activity prediction. The Wilcoxon signed-rank test[1] was applied to compare paired fold-wise performance.

**ROC_AUC**	**Fold 1**	**Fold 2**	**Fold 3**	**Fold 4**	**Fold 5**	**p-value**
XGBoost	0.9691	0.9702	0.9679	0.9728	0.9671	0.0625
ET	0.9670	0.9698	0.9673	0.9707	0.9651	

Table S2. Nested 5-fold cross-validation quality parameters of binary classification models predicting S2R active and inactive classes. For each metric, the mean value and standard deviation are reported.

Model	Descriptor	ROC_AUC	F1-score	Precision	Recall	MCC
	rdkit	0.882 ± 0.025	0.868 ± 0.024	0.947 ± 0.010	0.802 ± 0.039	0.506 ± 0.051
	mordred	0.883 ± 0.027	0.874 ± 0.019	0.949 ± 0.010	0.810 ± 0.033	0.520 ± 0.041
XGBoost	ECFP4	0.874 ± 0.041	0.869 ± 0.032	0.956 ± 0.018	0.797 ± 0.047	0.530 ± 0.082
	ECFP6	0.878 ± 0.039	0.872 ± 0.017	0.953 ± 0.011	0.804 ± 0.025	0.524 ± 0.047
	MACCS	0.872 ± 0.029	0.875 ± 0.020	0.952 ± 0.007	0.809 ± 0.029	0.529 ± 0.049
	rdkit	0.880 ± 0.034	0.871 ± 0.024	0.952 ± 0.007	0.803 ± 0.040	0.522 ± 0.042
	mordred	0.892 ± 0.030	0.878 ± 0.023	0.953 ± 0.010	0.815 ± 0.039	0.542 ± 0.050
ET	ECFP4	0.881 ± 0.043	0.879 ± 0.019	0.967 ± 0.016	0.807 ± 0.029	0.568 ± 0.057
	ECFP6	0.880 ± 0.040	0.871 ± 0.016	0.963 ± 0.014	0.796 ± 0.021	0.546 ± 0.052
	MACCS	0.876 ± 0.040	0.877 ± 0.024	0.955 ± 0.011	0.811 ± 0.035	0.540 ± 0.062
	rdkit	0.860 ± 0.033	0.869 ± 0.028	0.942 ± 0.013	0.809 ± 0.048	0.497 ± 0.050
	mordred	0.860 ± 0.042	0.860 ± 0.025	0.948 ± 0.018	0.787 ± 0.034	0.493 ± 0.077
KNN	ECFP4	0.849 ± 0.037	0.875 ± 0.026	0.922 ± 0.034	0.836 ± 0.061	0.452 ± 0.099
	ECFP6	0.843 ± 0.041	0.861 ± 0.038	0.933 ± 0.034	0.803 ± 0.078	0.463 ± 0.082
	MACCS	0.844 ± 0.040	0.866 ± 0.035	0.939 ± 0.013	0.805 ± 0.053	0.488 ± 0.080
	rdkit	0.878 ± 0.024	0.865 ± 0.021	0.956 ± 0.007	0.791 ± 0.037	0.521 ± 0.031
	mordred	0.882 ± 0.033	0.876 ± 0.026	0.948 ± 0.007	0.814 ± 0.041	0.524 ± 0.057
RF	ECFP4	0.884 ± 0.039	0.879 ± 0.022	0.968 ± 0.015	0.805 ± 0.033	0.569 ± 0.057
	ECFP6	0.877 ± 0.040	0.872 ± 0.022	0.964 ± 0.015	0.797 ± 0.032	0.551 ± 0.061
	MACCS	0.879 ± 0.040	0.882 ± 0.027	0.959 ± 0.017	0.816 ± 0.040	0.557 ± 0.075
	rdkit	0.873 ± 0.038	0.865 ± 0.042	0.950 ± 0.007	0.796 ± 0.068	0.514 ± 0.075
	mordred	0.882 ± 0.043	0.880 ± 0.033	0.953 ± 0.017	0.817 ± 0.050	0.544 ± 0.087
SVM	ECFP4	0.877 ± 0.047	0.872 ± 0.034	0.960 ± 0.017	0.799 ± 0.048	0.544 ± 0.092
	ECFP6	0.872 ± 0.043	0.870 ± 0.027	0.967 ± 0.017	0.790 ± 0.036	0.552 ± 0.075
	MACCS	0.866 ± 0.045	0.858 ± 0.033	0.956 ± 0.014	0.779 ± 0.050	0.512 ± 0.072

Table S3. Nested 5-fold cross-validation quality parameters of binary classification models predicting S1R selective and non-selective classes. For each metric, the mean value and standard deviation are reported.

Model	Descriptor	ROC_AUC	F1-score	Precision	Recall	MCC
XGBoost	rdkit	0.851 ± 0.091	0.402 ± 0.087	0.277 ± 0.067	0.741 ± 0.121	0.374 ± 0.108
	mordred	0.884 ± 0.033	0.396 ± 0.039	0.265 ± 0.029	0.789 ± 0.073	0.378 ± 0.050
	ECFP4	0.872 ± 0.038	0.429 ± 0.076	0.292 ± 0.072	0.841 ± 0.080	0.420 ± 0.078
	ECFP6	0.873 ± 0.026	0.397 ± 0.031	0.268 ± 0.029	0.778 ± 0.032	0.378 ± 0.028
	MACCS	0.862 ± 0.038	0.392 ± 0.079	0.269 ± 0.059	0.728 ± 0.131	0.363 ± 0.100
ET	rdkit	0.857 ± 0.063	0.456 ± 0.105	0.337 ± 0.099	0.728 ± 0.131	0.426 ± 0.121
	mordred	0.892 ± 0.045	0.434 ± 0.101	0.319 ± 0.092	0.691 ± 0.099	0.398 ± 0.114
	ECFP4	0.885 ± 0.045	0.455 ± 0.052	0.330 ± 0.048	0.741 ± 0.082	0.428 ± 0.059
	ECFP6	0.881 ± 0.047	0.425 ± 0.103	0.312 ± 0.078	0.678 ± 0.175	0.389 ± 0.124
	MACCS	0.877 ± 0.047	0.420 ± 0.073	0.308 ± 0.063	0.666 ± 0.098	0.381 ± 0.085
KNN	rdkit	0.856 ± 0.068	0.395 ± 0.088	0.269 ± 0.059	0.740 ± 0.175	0.369 ± 0.119
	mordred	0.874 ± 0.040	0.381 ± 0.084	0.253 ± 0.061	0.776 ± 0.152	0.360 ± 0.108
	ECFP4	0.865 ± 0.032	0.205 ± 0.043	0.115 ± 0.027	1.000 ± 0.000	0.170 ± 0.091
	ECFP6	0.844 ± 0.095	0.253 ± 0.090	0.149 ± 0.060	0.964 ± 0.053	0.218 ± 0.147
	MACCS	0.859 ± 0.041	0.395 ± 0.087	0.283 ± 0.073	0.665 ± 0.125	0.355 ± 0.103
RF	rdkit	0.865 ± 0.073	0.430 ± 0.085	0.302 ± 0.065	0.753 ± 0.153	0.405 ± 0.109
	mordred	0.890 ± 0.038	0.456 ± 0.058	0.337 ± 0.048	0.716 ± 0.115	0.425 ± 0.070
	ECFP4	0.890 ± 0.031	0.424 ± 0.073	0.302 ± 0.071	0.728 ± 0.037	0.394 ± 0.077
	ECFP6	0.879 ± 0.048	0.409 ± 0.097	0.300 ± 0.068	0.653 ± 0.188	0.370 ± 0.121
	MACCS	0.870 ± 0.054	0.427 ± 0.112	0.310 ± 0.094	0.703 ± 0.137	0.393 ± 0.132
SVM	rdkit	0.822 ± 0.077	0.369 ± 0.110	0.259 ± 0.096	0.678 ± 0.175	0.331 ± 0.131
	mordred	0.877 ± 0.044	0.452 ± 0.105	0.357 ± 0.142	0.703 ± 0.143	0.423 ± 0.110
	ECFP4	0.881 ± 0.027	0.471 ± 0.114	0.387 ± 0.127	0.629 ± 0.127	0.429 ± 0.122
	ECFP6	0.880 ± 0.033	0.452 ± 0.101	0.372 ± 0.118	0.605 ± 0.069	0.406 ± 0.106
	MACCS	0.833 ± 0.060	0.404 ± 0.109	0.374 ± 0.281	0.616 ± 0.165	0.376 ± 0.138

Table S4. Nested 5-fold cross-validation quality parameters of binary classification models predicting S2R selective and non-selective classes. For each metric, the mean value and standard deviation are reported.

Model	Descriptor	ROC_AUC	F1-score	Precision	Recall	MCC
XGBoost	rdkit	0.845 ± 0.042	0.442 ± 0.047	0.317 ± 0.047	0.737 ± 0.027	0.380 ± 0.054
	mordred	0.856 ± 0.035	0.446 ± 0.025	0.317 ± 0.011	0.755 ± 0.098	0.389 ± 0.043
	ECFP4	0.834 ± 0.078	0.455 ± 0.069	0.323 ± 0.055	0.773 ± 0.093	0.400 ± 0.091
	ECFP6	0.849 ± 0.053	0.480 ± 0.033	0.346 ± 0.028	0.790 ± 0.082	0.430 ± 0.046
	MACCS	0.846 ± 0.054	0.416 ± 0.026	0.286 ± 0.031	0.782 ± 0.105	0.361 ± 0.032
ET	rdkit	0.863 ± 0.052	0.521 ± 0.024	0.392 ± 0.025	0.781 ± 0.052	0.472 ± 0.028
	mordred	0.879 ± 0.055	0.524 ± 0.019	0.397 ± 0.031	0.781 ± 0.052	0.476 ± 0.020
	ECFP4	0.873 ± 0.064	0.533 ± 0.056	0.412 ± 0.045	0.755 ± 0.077	0.480 ± 0.070
	ECFP6	0.867 ± 0.057	0.543 ± 0.057	0.440 ± 0.051	0.711 ± 0.077	0.486 ± 0.069
	MACCS	0.864 ± 0.041	0.471 ± 0.031	0.347 ± 0.038	0.747 ± 0.111	0.415 ± 0.043
KNN	rdkit	0.821 ± 0.067	0.404 ± 0.033	0.274 ± 0.025	0.790 ± 0.120	0.349 ± 0.053
	mordred	0.839 ± 0.046	0.434 ± 0.053	0.298 ± 0.038	0.808 ± 0.124	0.385 ± 0.076
	ECFP4	0.833 ± 0.039	0.282 ± 0.079	0.169 ± 0.058	0.939 ± 0.039	0.193 ± 0.131
	ECFP6	0.848 ± 0.061	0.265 ± 0.035	0.154 ± 0.023	0.974 ± 0.058	0.190 ± 0.076
	MACCS	0.834 ± 0.032	0.389 ± 0.048	0.258 ± 0.038	0.806 ± 0.093	0.334 ± 0.066
RF	rdkit	0.858 ± 0.039	0.496 ± 0.033	0.377 ± 0.036	0.729 ± 0.033	0.438 ± 0.038
	mordred	0.880 ± 0.037	0.529 ± 0.038	0.399 ± 0.055	0.769 ± 0.034	0.474 ± 0.039
	ECFP4	0.865 ± 0.065	0.526 ± 0.064	0.417 ± 0.065	0.720 ± 0.047	0.469 ± 0.074
	ECFP6	0.867 ± 0.066	0.524 ± 0.079	0.411 ± 0.071	0.729 ± 0.093	0.467 ± 0.096
	MACCS	0.866 ± 0.039	0.477 ± 0.046	0.350 ± 0.051	0.764 ± 0.089	0.424 ± 0.055
SVM	rdkit	0.827 ± 0.055	0.439 ± 0.040	0.311 ± 0.031	0.755 ± 0.103	0.381 ± 0.058
	mordred	0.870 ± 0.050	0.500 ± 0.035	0.372 ± 0.023	0.772 ± 0.099	0.450 ± 0.049
	ECFP4	0.877 ± 0.057	0.484 ± 0.065	0.351 ± 0.055	0.790 ± 0.112	0.435 ± 0.084
	ECFP6	0.884 ± 0.058	0.545 ± 0.078	0.425 ± 0.087	0.772 ± 0.078	0.496 ± 0.091
	MACCS	0.823 ± 0.050	0.468 ± 0.081	0.404 ± 0.150	0.624 ± 0.118	0.403 ± 0.093

Tables of nested 5-fold cross-validation quality metrics for multiclass classification models evaluated for the single-step multiclassification workflow.

Table S5. Nested 5-fold cross-validation quality parameters of 3-multiclass classification models predicting S2R selective, non-selective, and inactive classes. For each metric, the mean value and standard deviation are reported.

Model	Descriptor	ROC_AUC	F1-score	Precision	Recall	MCC
XGBoost	rdkit	0.933 ± 0.013	0.829 ± 0.032	0.863 ± 0.028	0.808 ± 0.035	0.675 ± 0.056
	mordred	0.938 ± 0.016	0.839 ± 0.024	0.869 ± 0.025	0.822 ± 0.024	0.695 ± 0.040
	ECFP4	0.914 ± 0.017	0.802 ± 0.027	0.836 ± 0.021	0.782 ± 0.032	0.630 ± 0.046
	ECFP6	0.917 ± 0.014	0.805 ± 0.022	0.835 ± 0.021	0.786 ± 0.027	0.634 ± 0.040
	MACCS	0.916 ± 0.019	0.818 ± 0.029	0.854 ± 0.027	0.798 ± 0.029	0.659 ± 0.046
ET	rdkit	0.934 ± 0.010	0.841 ± 0.019	0.869 ± 0.014	0.825 ± 0.021	0.699 ± 0.032
	mordred	0.941 ± 0.012	0.852 ± 0.026	0.881 ± 0.023	0.836 ± 0.025	0.718 ± 0.042
	ECFP4	0.918 ± 0.016	0.822 ± 0.029	0.836 ± 0.029	0.813 ± 0.030	0.667 ± 0.053
	ECFP6	0.913 ± 0.017	0.819 ± 0.030	0.833 ± 0.031	0.810 ± 0.031	0.663 ± 0.055
	MACCS	0.919 ± 0.012	0.825 ± 0.015	0.854 ± 0.012	0.809 ± 0.016	0.672 ± 0.027
KNN	rdkit	0.894 ± 0.016	0.741 ± 0.021	0.835 ± 0.022	0.701 ± 0.013	0.538 ± 0.024
	mordred	0.902 ± 0.010	0.732 ± 0.060	0.860 ± 0.023	0.683 ± 0.064	0.542 ± 0.072
	ECFP4	0.886 ± 0.021	0.695 ± 0.079	0.771 ± 0.043	0.711 ± 0.059	0.511 ± 0.093
	ECFP6	0.873 ± 0.023	0.471 ± 0.083	0.716 ± 0.051	0.568 ± 0.045	0.286 ± 0.093
	MACCS	0.885 ± 0.017	0.751 ± 0.032	0.830 ± 0.016	0.723 ± 0.034	0.562 ± 0.044
RF	rdkit	0.936 ± 0.014	0.844 ± 0.027	0.876 ± 0.022	0.826 ± 0.029	0.704 ± 0.046
	mordred	0.940 ± 0.014	0.845 ± 0.023	0.878 ± 0.024	0.826 ± 0.022	0.704 ± 0.038
	ECFP4	0.917 ± 0.016	0.820 ± 0.035	0.835 ± 0.035	0.811 ± 0.036	0.664 ± 0.063
	ECFP6	0.914 ± 0.020	0.818 ± 0.037	0.831 ± 0.037	0.810 ± 0.038	0.662 ± 0.067
	MACCS	0.918 ± 0.015	0.822 ± 0.020	0.851 ± 0.017	0.806 ± 0.020	0.667 ± 0.032
	rdkit	0.917 ± 0.014	0.812 ± 0.026	0.846 ± 0.022	0.793 ± 0.027	0.647 ± 0.043
	mordred	0.920 ± 0.018	0.803 ± 0.024	0.841 ± 0.030	0.784 ± 0.024	0.636 ± 0.042

SVM	ECFP4	0.901 ± 0.024	0.811 ± 0.045	0.835 ± 0.037	0.797 ± 0.050	0.648 ± 0.078
	ECFP6	0.905 ± 0.020	0.799 ± 0.048	0.819 ± 0.043	0.795 ± 0.042	0.636 ± 0.073
	MACCS	0.898 ± 0.026	0.797 ± 0.031	0.822 ± 0.038	0.788 ± 0.028	0.629 ± 0.053

Table S6. Nested 5-fold cross-validation quality parameters of 3-multiclass classification models predicting S1R selective, non-selective, and inactive classes. For each metric, the mean value and standard deviation are reported.

Model	Descriptor	ROC_AUC	F1-score	Precision	Recall	MCC
XGBoost	rdkit	0.854 ± 0.026	0.708 ± 0.014	0.771 ± 0.014	0.688 ± 0.013	0.477 ± 0.019
	mordred	0.864 ± 0.030	0.736 ± 0.019	0.798 ± 0.014	0.716 ± 0.021	0.527 ± 0.023
	ECFP4	0.850 ± 0.035	0.727 ± 0.020	0.786 ± 0.034	0.709 ± 0.018	0.509 ± 0.055
	ECFP6	0.858 ± 0.033	0.738 ± 0.022	0.802 ± 0.023	0.718 ± 0.022	0.534 ± 0.035
	MACCS	0.852 ± 0.026	0.730 ± 0.012	0.802 ± 0.017	0.707 ± 0.014	0.522 ± 0.016
ET	rdkit	0.864 ± 0.030	0.747 ± 0.016	0.795 ± 0.024	0.732 ± 0.018	0.571 ± 0.037
	mordred	0.870 ± 0.031	0.766 ± 0.020	0.811 ± 0.028	0.751 ± 0.018	0.716 ± 0.045
	ECFP4	0.854 ± 0.040	0.755 ± 0.031	0.802 ± 0.030	0.740 ± 0.033	0.629 ± 0.054
	ECFP6	0.853 ± 0.040	0.757 ± 0.028	0.803 ± 0.032	0.743 ± 0.028	0.622 ± 0.056
	MACCS	0.846 ± 0.028	0.727 ± 0.033	0.782 ± 0.021	0.709 ± 0.038	0.535 ± 0.048
KNN	rdkit	0.826 ± 0.038	0.692 ± 0.028	0.768 ± 0.029	0.663 ± 0.031	0.442 ± 0.046
	mordred	0.824 ± 0.042	0.708 ± 0.037	0.781 ± 0.037	0.681 ± 0.040	0.470 ± 0.064
	ECFP4	0.808 ± 0.060	0.602 ± 0.072	0.759 ± 0.046	0.553 ± 0.088	0.348 ± 0.108
	ECFP6	0.785 ± 0.064	0.572 ± 0.088	0.759 ± 0.048	0.530 ± 0.102	0.348 ± 0.135
	MACCS	0.797 ± 0.052	0.669 ± 0.047	0.771 ± 0.042	0.628 ± 0.053	0.404 ± 0.067
RF	rdkit	0.859 ± 0.030	0.736 ± 0.026	0.787 ± 0.025	0.721 ± 0.029	0.520 ± 0.046
	mordred	0.866 ± 0.028	0.755 ± 0.018	0.802 ± 0.023	0.739 ± 0.017	0.546 ± 0.034
	ECFP4	0.859 ± 0.041	0.756 ± 0.026	0.803 ± 0.032	0.739 ± 0.025	0.548 ± 0.051
	ECFP6	0.850 ± 0.045	0.751 ± 0.031	0.800 ± 0.037	0.736 ± 0.031	0.544 ± 0.063
	MACCS	0.847 ± 0.025	0.727 ± 0.025	0.793 ± 0.031	0.706 ± 0.024	0.514 ± 0.051
SVM	rdkit	0.853 ± 0.031	0.711 ± 0.011	0.782 ± 0.031	0.690 ± 0.010	0.494 ± 0.044
	mordred	0.856 ± 0.032	0.713 ± 0.023	0.784 ± 0.031	0.695 ± 0.020	0.502 ± 0.046
	ECFP4	0.853 ± 0.043	0.746 ± 0.043	0.781 ± 0.050	0.732 ± 0.043	0.510 ± 0.089
	ECFP6	0.854 ± 0.030	0.714 ± 0.019	0.785 ± 0.026	0.696 ± 0.018	0.506 ± 0.037
	MACCS	0.830 ± 0.047	0.696 ± 0.018	0.765 ± 0.032	0.675 ± 0.016	0.461 ± 0.052

Tables of nested 5-fold cross-validation quality metrics for regression models evaluated for the two-step regression workflow.

Table S7. Nested 5-fold cross-validation quality parameters of regression models predicting S1R pActivity values. For each metric, the mean value and standard deviation are reported.

ML algorithm	Descriptor	RMSE	R^2 score
XGBoost	rdkit	0.673 ± 0.015	0.736 ± 0.009
	mordred	0.654 ± 0.014	0.750 ± 0.008
	ECFP4	0.671 ± 0.014	0.737 ± 0.010
	ECFP6	0.673 ± 0.020	0.736 ± 0.013
	maccs	0.707 ± 0.008	0.708 ± 0.006
ET	rdkit	0.706 ± 0.008	0.708 ± 0.005
	mordred	0.682 ± 0.008	0.728 ± 0.004
	ECFP4	0.689 ± 0.013	0.722 ± 0.011
	ECFP6	0.689 ± 0.016	0.723 ± 0.007
	maccs	0.712 ± 0.011	0.704 ± 0.008
KNN	rdkit	0.777 ± 0.016	0.647 ± 0.017
	mordred	0.749 ± 0.023	0.672 ± 0.024
	ECFP4	0.816 ± 0.019	0.611 ± 0.013
	ECFP6	0.783 ± 0.023	0.642 ± 0.019
	maccs	0.808 ± 0.022	0.618 ± 0.029
RF	rdkit	0.685 ± 0.011	0.726 ± 0.004
	mordred	0.678 ± 0.011	0.732 ± 0.004
	ECFP4	0.680 ± 0.015	0.730 ± 0.006
	ECFP6	0.687 ± 0.013	0.724 ± 0.006
	maccs	0.715 ± 0.014	0.701 ± 0.011
SVM	rdkit	0.716 ± 0.018	0.701 ± 0.016
	mordred	0.683 ± 0.019	0.727 ± 0.014
	ECFP4	0.737 ± 0.025	0.682 ± 0.019

	ECFP6	0.726 ± 0.016	0.692 ± 0.009
	maccs	0.737 ± 0.020	0.682 ± 0.018

Table S8. Nested 5-fold cross-validation quality parameters of regression models predicting S2R pActivity values. For each metric, the mean value and standard deviation are reported.

ML algorithm	Descriptor	RMSE	R² score
XGBoost	rdkit	1.171 ± 0.082	0.526 ± 0.034
	mordred	1.178 ± 0.099	0.521 ± 0.051
	ECFP4	1.188 ± 0.040	0.511 ± 0.027
	ECFP6	1.214 ± 0.048	0.490 ± 0.023
	maccs	1.202 ± 0.098	0.501 ± 0.049
ET	rdkit	1.185 ± 0.081	0.515 ± 0.033
	mordred	1.149 ± 0.095	0.544 ± 0.043
	ECFP4	1.164 ± 0.077	0.532 ± 0.029
	ECFP6	1.178 ± 0.082	0.521 ± 0.034
	maccs	1.192 ± 0.097	0.509 ± 0.050
KNN	rdkit	1.277 ± 0.086	0.436 ± 0.042
	mordred	1.246 ± 0.088	0.463 ± 0.044
	ECFP4	1.327 ± 0.087	0.391 ± 0.055
	ECFP6	1.247 ± 0.098	0.461 ± 0.059
	maccs	1.364 ± 0.109	0.354 ± 0.092
RF	rdkit	1.178 ± 0.094	0.521 ± 0.041
	mordred	1.168 ± 0.094	0.529 ± 0.043
	ECFP4	1.185 ± 0.038	0.514 ± 0.027
	ECFP6	1.202 ± 0.047	0.500 ± 0.030
	maccs	1.206 ± 0.094	0.497 ± 0.051
	rdkit	1.185 ± 0.103	0.514 ± 0.058
	mordred	1.159 ± 0.104	0.535 ± 0.060

SVM	ECFP4	1.232 ± 0.090	0.475 ± 0.048
	ECFP6	1.228 ± 0.085	0.479 ± 0.036
	maccs	1.234 ± 0.074	0.474 ± 0.030

Confusion matrices were generated to assess the performance of the ET multiclassification models across 5-fold classical cross-validation in predicting activity and selectivity profiles for S1R and S2R.

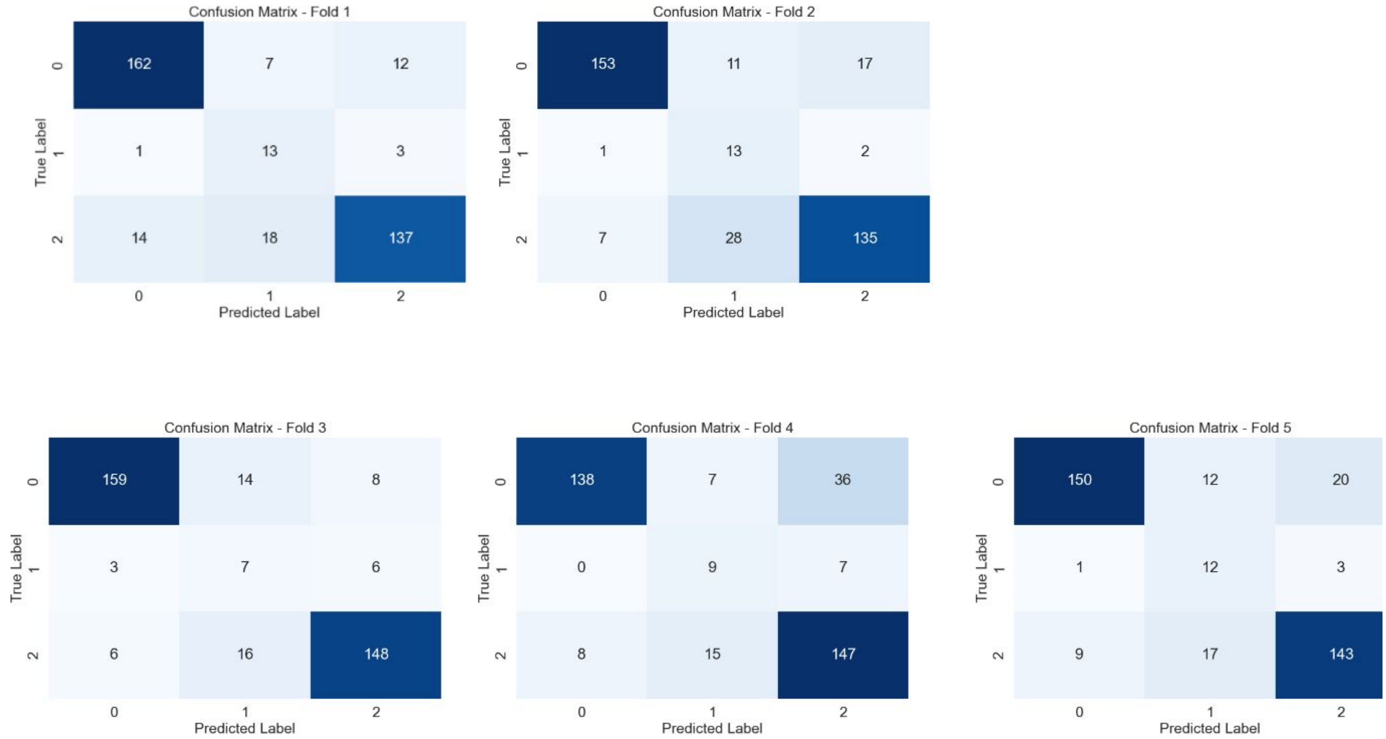

Figure S2. Confusion matrices generated across the 5-fold in classical cross-validation to predict selective, non-selective, and inactive compounds for S1R. In the confusion matrices, inactives are labeled as “0”, selective molecules as “1”, and non-selective compounds as “2”. Results along the diagonal of the confusion matrix indicate the data was predicted correctly.

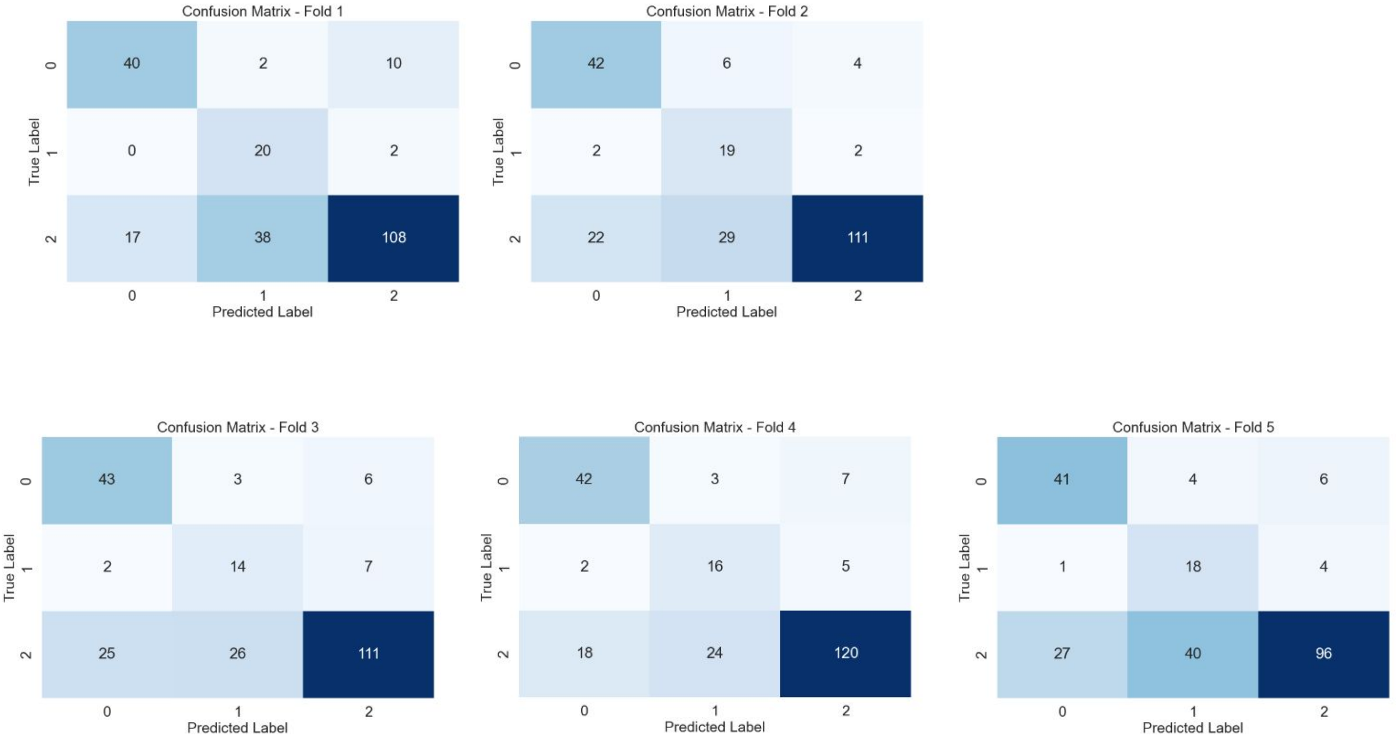

Figure S3. Confusion matrices generated across the 5-fold in classical cross-validation to predict selective, non-selective, and inactive compounds for S2R.In confusion matrices, inactives are labeled as “0”, selective molecules as “1”, and non-selective compounds as “2”. Results along the diagonal of the confusion matrix indicate the data was predicted correctly.

Table S9. Classical 5-fold cross-validation quality parameters of baseline models predicting S1R selectivity labels. For each metric, the mean value and standard deviation are reported.

Model	ROC_AUC	F1-score	Precision	Recall	MCC
Logistic Regression	0.704 ± 0.052	0.473 ± 0.068	0.538 ± 0.076	0.444 ± 0.119	0.441 ± 0.064
1-Nearest Neighbor	0.713 ± 0.077	0.477 ± 0.134	0.494 ± 0.118	0.468 ± 0.155	0.435 ± 0.139
Decision Tree (depth=3)	0.608 ± 0.051	0.303 ± 0.114	0.428 ± 0.094	0.260 ± 0.140	0.275 ± 0.093
LogReg on SLogP only	0.499 ± 0.001	0.000 ± 0.000	0.000 ± 0.000	0.000 ± 0.000	-0.004 ± 0.008
Dummy Most Frequent	0.500 ± 0.000	0.000 ± 0.000	0.000 ± 0.000	0.000 ± 0.000	0.000 ± 0.000
Dummy Uniform	0.486 ± 0.069	0.162 ± 0.031	0.086 ± 0.016	0.480 ± 0.106	0.010 ± 0.046

Table S10. Classical 5-fold cross-validation quality parameters of baseline models predicting S2R selectivity labels. For each metric, the mean value and standard deviation are reported.

Model	ROC_AUC	F1-score	Precision	Recall	MCC
Logistic Regression	0.731 ± 0.048	0.544 ± 0.065	0.601 ± 0.076	0.509 ± 0.104	0.497 ± 0.070
1-Nearest Neighbor	0.768 ± 0.030	0.582 ± 0.058	0.583 ± 0.118	0.596 ± 0.061	0.530 ± 0.073
Decision Tree (depth=3)	0.669 ± 0.041	0.477 ± 0.073	0.692 ± 0.167	0.360 ± 0.090	0.464 ± 0.088
LogReg on SLogP only	0.500 ± 0.000	0.000 ± 0.000	0.000 ± 0.000	0.000 ± 0.000	0.000 ± 0.000
Dummy Most Frequent	0.500 ± 0.000	0.000 ± 0.000	0.000 ± 0.000	0.000 ± 0.000	0.000 ± 0.000
Dummy Uniform	0.476 ± 0.044	0.181 ± 0.027	0.107 ± 0.018	0.587 ± 0.073	0.008 ± 0.066

Table S11. Classical 5-fold cross-validation quality parameters of Dummy Regressor predicting pActivity values. For each metric, the mean value and standard deviation are reported.

Model	Target	RMSE	R^2 score
Dummy Regressor (Median)	S1R	1.314± 0.033	-0.01± 0.009
Dummy Regressor (Median)	S2R	1.728 ± 0.064	-0.031 ± 0.01

Table S22. Feature profiles of four S1R-selective ligands, each characterized by their top 15 most important molecular descriptors as identified by the ET multiclass model.

cmpd	nBase	NsssN	NaaN	SssCH2	SMR_VSA3	PEOE_VSA7	BalabanJ	EState_VSA4
1	1	1	0	4.05	10.22	101.76	1.42	31.04
2	1	0	0	3.17	10.63	48.86	1.49	25.20
3	1	1	0	14.05	4.90	62.62	1.36	25.18
4	1	1	1	8.48	20.00	81.32	1.16	79.83
cmpd	NaaCH	NssCH2	NaasN	SLogP	CIC0	SaasC	nRing	
1	7	8	0	6.22	4.47	1.16	4	
2	8	4	0	3.29	3.95	3.63	2	
3	4	11	0	4.88	4.68	2.83	4	
4	9	10	1	5.21	4.49	3.81	5	

Table S33. Feature profiles of four S2R-selective ligands, each characterized by their top 15 most important molecular descriptors as identified by the ET multiclass model.

cmpd	n10FARing	n10FHRing	nBase	AATS5d	SlogP_VSA11	AATS0dv	NaaN	AATS6d
**5**	1	1	1	2.46	11.50	7.12	0	2.40
**6**	0	0	1	2.37	0.00	6.50	0	2.76
**7**	1	1	1	2.40	23.00	7.15	0	2.22
**8**	0	0	1	2.30	0.00	7.47	0	2.39

cmpd	n10FRing	NssO	n6AHRing	PEOE_VSA13	AATS7d	C1SP2	PEOE_VSA11
**5**	1	2	1	5.91	2.24	1	17.26
**6**	0	0	1	0.00	2.83	0	0.00
**7**	1	4	1	5.91	2.11	1	23.00
**8**	0	0	1	0.00	2.45	0	5.58

References

(1) Woolson, RF. Wilcoxon Signed-Rank Test. In Wiley Encyclopedia of Clinical Trials, D'Agostino RB, Sullivan L, Massaro J, eds. 2008. DOI:10.1002/9780471462422.eoct979
